# Protective effect of alogliptin against cyclophosphamide-induced lung toxicity in rats: Impact on PI3K/Akt/FoxO1 pathway and downstream inflammatory cascades

**DOI:** 10.1007/s00441-022-03593-1

**Published:** 2022-02-02

**Authors:** Amira Ebrahim Alsemeh, Doaa M. Abdullah

**Affiliations:** 1 Human Anatomy and Embryology Department, Faculty of Medicine, Zagazig University, Zagazig, Egypt; 2 Clinical Pharmacology Department, Faculty of Medicine, Zagazig University, Zagazig, Egypt

**Keywords:** Alogliptin, Apoptosis, Cyclophosphamide, Fibrosis, Oxidative stress

## Abstract

Cyclophosphamide (CP)-induced lung toxicity is a remaining obstacle against the beneficial use of this chemotherapeutic agent. More considerations were given to the role of Alogliptin (ALO) in ameliorating CP-induced toxicities in many tissues. We designed this study to clarify the protective potential of ALO against CP-induced lung toxicity in rats. ALO was administered for 7 days. Single-dose CP was injected on the 2nd day (200 mg/kg: i.p.) to induce lung toxicity. Rats were divided into four groups: control, ALO-treated, CP-treated and ALO + CP-treated group. Leucocytic count, total proteins, LDH activity, TNF-α, and IL-6 were estimated in the bronchoalveolar lavage fluid (BALF). The oxidative/antioxidants (MDA, Nrf2, TAO and GSH), inflammatory (NFκB), fibrotic (TGF-β1) and apoptotic (PI3K/Akt/FoxO1) markers in pulmonary homogenates were biochemically evaluated. Rat lung sections were examined histologically (light and electron microscopic examination) and immunohistochemically (for iNOS and CD68 positive alveolar macrophages). CP significantly increased oxidative stress, inflammation, fibrosis, and apoptosis markers as well as deteriorated the histopathological pulmonary architecture. These hazardous effects were significantly ameliorated by ALO treatment. ALO protected against CP-induced lung toxicity by mitigating the oxidative, inflammatory and fibrotic impacts making it a promising pharmacological therapy for mitigating CP-induced lung toxicity.

**Graphical abstract:**

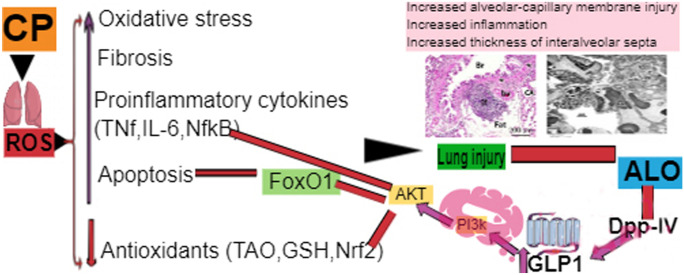

## Introduction

Cyclophosphamide (CP) is a very potent widely used alkylating agent. It is used as antineoplastic and immunosuppressive drug (Moignet et al. 2013). It is metabolized by liver enzymes to phosphoramide mustard and acrolein. The active anticancer agent acting on DNA causing cancer cell mortality is phosphoramide, whereas acrolein is the CP-toxic metabolite leading to toxicity (Sun et al. 2014a, b).

CP is associated with genotoxicity and numerous hazardous impacts on numerous organs due to its capability to stimulate oxidative burden with consequent cell death (El-Emam 2020). Among these toxicities are cardiotoxicity (Iqubal et al. 2019), hepatotoxicity (Abdelfattah-Hassan et al. 2019), nephrotoxicity (Sharma et al. 2017), in addition to lung toxicity (El-kashef 2018; Abdel-Latif et al. 2020).

Numerous earlier studies denoted that the hazardous toxicity of CP is caused by acrolein which leads to production of reactive oxygen species (ROS) leading to perturbing the cellular antioxidant defenses (El-kashef 2018). This overwhelming oxidative stress status causes increased pro-inflammatory cytokines production and encouragement of several inflammatory and fibrotic signaling pathways (Sun et al. 2014a, b).

Synchronously, the phosphoinositide 3-kinase (PI3K) and protein kinase B (Akt) signaling axis applies a crucial function in controlling cellular proliferation in response to various stimuli (Liu et al. 2019) by mitigating fork head box protein O1 (FoxO1) (Zeng et al. 2019; Wang et al. 2019). These findings motivate researchers to discover therapeutic strategies that can pursue PI3K/Akt /FoxO1 pathway to ameliorate cellular damage.

Alogliptin (ALO) is an antidiabetic drug that acts by inhibiting dipeptidyl peptidase 4 (DDP-IV) enzyme leading to upregulating glucagon-like peptide 1 (GLP-1) (Flatt 2008). Remarkably, gliptins are continuously investigated for novel biological actions as they can target numerous signaling axis’s (Kim and Schuppan 2012).

Previously more considerations were given to the role of ALO in ameliorating CP-induced toxicities in many tissues due to its antioxidant, anti-inflammatory in addition to antifibrotic effect (Kabel 2018; Salama et al. 2020a, b, c). Nevertheless, obvious studies that fully illuminate the molecular mechanisms of these defensive effects are deficient. Additionally, its potential mechanism in preventing lung injury induced by CP remains elusive.

Many earlier studies reported that enhanced GLP-1 concentrations can activate the downstream PI3K/Akt signaling axis. This subsequently phosphorylate several substrates, one of them is FoxO1 (Athauda and Foltynie 2016; Huang and Lin 2016). Therefore, our current research intended to clarify the possible curative use of ALO to protect against CP-induced lung toxicity through targeting the PI3K/Akt/FoxO1 axis as well as its influence on lung oxidative stress, fibrosis, and inflammatory markers, as well as lung histopathological architecture.

Based on the above observations, we hypothesized that ALO administration in a CP-induced lung toxicity animal model would protect against CP-induced lung toxicity through targeting the PI3K/Akt/FoxO1 axis as well ameliorating lung oxidative stress, fibrosis, and inflammatory markers induced by CP injection.

## Materials and methods

### Chemicals

Alogliptin (Sigma-Aldrich, MO) was given for 7 days at dose of 20 mg/kg/day p.o. ALO was suspended in 0.5% carboxymethyl cellulose (CMC) sodium to attain a last concentration of 2 mg/ml (Kabel 2018). Cyclophosphamide (CP) (Endoxan®, Baxter AG, Switzerland) was given as a sole-dose injection (200 mg/kg; i.p.) on day 2 to provoke acute lung toxicity. We dissolved CP in normal saline (0.9%) to a attain 2% concentration (20 mg/ml) (Caglayan et al. 2018). All other reagents and chemicals were commercially available.

### Animals

Forty-eight adult male 8-week-old Wistar rats (180 ± 20 g) were purchased from the animal house of Zagazig Scientific and Medical Research Centre (ZSMRC). Animals were allowed a one-week acclimatization period before beginning the study. They were put in standard polypropylene cages (3 rats per cage) at the animal facility of the Faculty of Medicine, Zagazig University. Rats were allowed free access to a normal pellet diet and tap water all through the study time. They were maintained under standard temperature (22 ± 2 °C) and relative humidity (55 ± 5%), with a 12-light/12-dark cycle. All experimental procedures and animal husbandry complies the ARRIVE guidelines (Kilkenny et al. 2010). Also following the international guidelines for the care and use of laboratory animals (Clark et al. 1997) and were revised and approved by the Zagazig University Institutional Animal Care Unit Committee, Zagazig University, Zagazig, Egypt, with approval number (ZU-IACUC/4/F/2020).

### Experimental groups

**Fig. 1:**
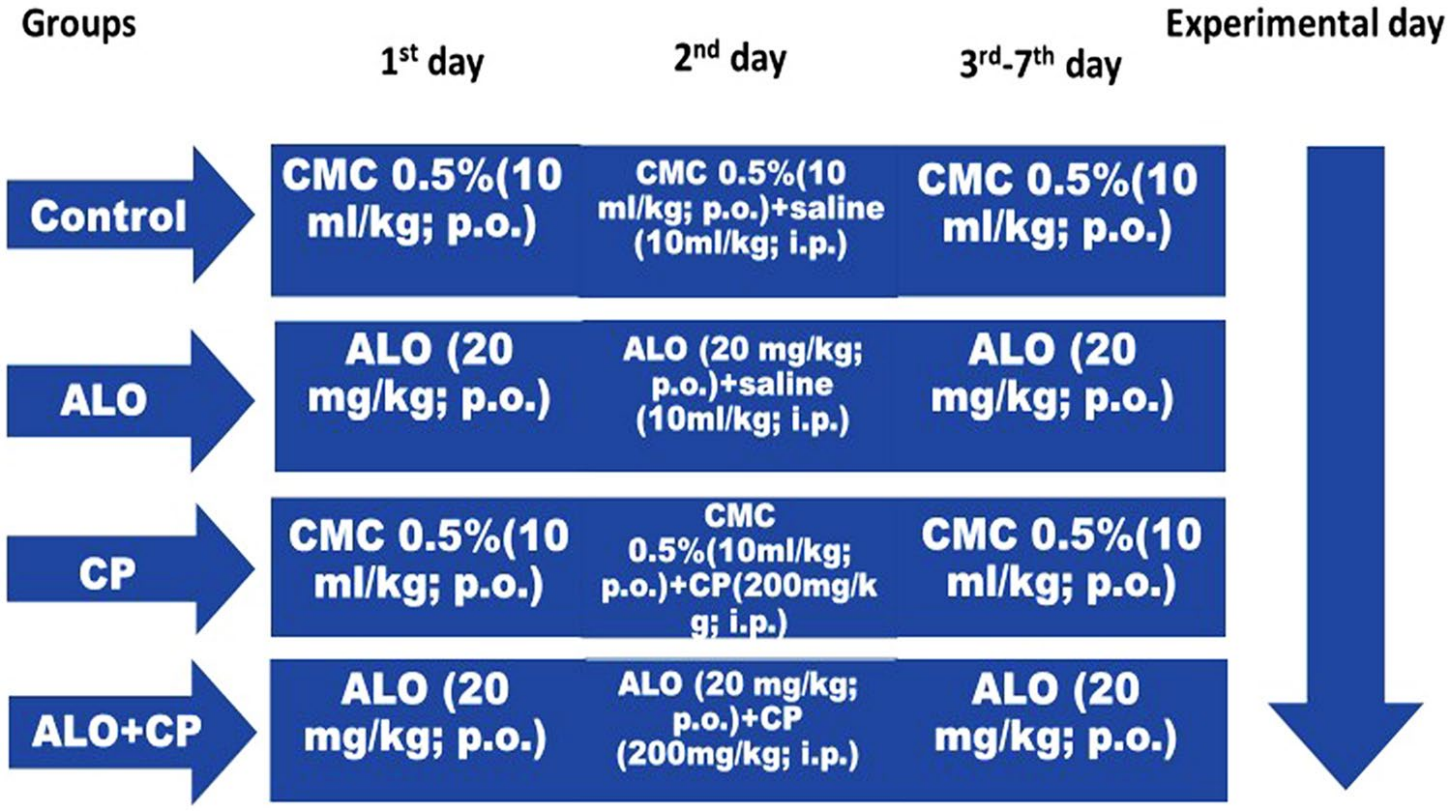
Experimental design. ALO: alogliptin; CMC: carboxymethylcellulose; CP: cyclophosphamide; i.p.: intraperitoneal; p.o.: per oral

The rats were randomly divided into four groups (n = 12/group) as following (Fig. 1):

#### Control group

Rats were administered CMC (0.5%, 10 ml/kg, orally); for seven days. Single dose saline (10 ml/kg.) was injected intraperitoneally on 2^nd^ day.

#### ALO group

Rats were administered ALO (20 mg/kg; orally) (Kabel 2018) for seven days. Single dose saline (10 ml/kg.) was injected intraperitoneally on 2^nd^ day.

#### CP group

Rats were administered CMC (0.5%, 10 ml/kg. orally) for seven days, and CP (200 mg/kg) was injected intraperitoneally on 2^nd^ day (Caglayan et al. 2018; Salama et al. 2020a).

#### ALO + CP group

Rats were administered ALO (20 mg/kg; orally) for seven days, and CP (200 mg/kg) was injected intraperitoneally on 2^nd^ day.

### Experimental design

#### Tissue and blood gathering

On 8th day, after overnight fasting a blood sample was obtained from rat tail and was used for measuring fasting blood glucose level.

Afterwards, sample tissues collection was done. Rats were anesthetized with a mixture of intraperitoneal ketamine hydrochloride (50 mg/kg) plus xylazine (5 mg/kg) (Salama et al. 2020a).

Six rats from each group were used to extract the broncho-alveolar lavage fluid (BALF).

The other six rats were exposed to cervical dislocation and the two lungs were removed and cleansed with ice-cold saline.

Afterwards, the left lungs were homogenized in isotonic saline to achieve 10% homogenate, and the supernatant was stored to be used for biochemical laboratory analysis.

Meanwhile, one lobe of right lung was fixed by instillation of fixative (10% neutral buffered formalin, pH 7.4) through syringes which were inserted into the airways of this lung lobe. Then the lung lobe was immersed and kept for 72 h in 10% neutral buffered formalin and processed for paraffin block rapidly to be processed for histopathological and immunohistochemical examination by light microscope (Bancroft and Layton 2012).

The other lobe of the right lung was fixed by instillation 2% glutaraldehyde in 0.085 M sodium cacodylate buffer (pH 7.4) through syringes which were inserted into the airways of lung lobe. The fixative was instilled quickly enough to achieve rapid filling of lung tissue while the lung was closely observed to avoid overdistension. This fixative is sufficiently rapid that the alveolar tissue becomes rigid. Then the lobe was divided into small pieces and promptly fixed in 2.5% glutaraldehyde buffer for preparation of semithin sections (examined by light microscope) and ultrathin section (examined by electron microscope) (Vlahovic et al. 1999).

#### Gathering of the bronchoalveolar lavage fluid (BALF)

After exposing the thoracic cavity, a cannula (size: 24G, flow rate: 16 mL/min) was inserted into the trachea to attain the BALF.

We made lavage for rats’ lungs was for three times. In each time lavage, 3 mL saline was injected into the lung through these tracheal cannulas and the saline was kept for 30 s in each lung. Afterwards the saline was aspirated. Finally, we attained about 9 mL of saline (BALF). Finally, the BALF recovery rates must exceed 80%. Then the gathered BALF was centrifugated for 10 min at 2000 rpm, 4 °C, and the supernatant was used for further biochemical laboratory analyses. Meanwhile, for estimation of total and differential cell counts, cell pellets were resuspended in 0.5 ml sterile saline (Verma et al. 2013).

#### Evaluation of total and differential leucocytic number

We used a hemocytometer to count the total numbers of leucocytic cells in the BALF pellets. For differential leucocytic cell counts, smear slides were organized and then stained with Giemsa solution (Verma et al. 2013). Differential counts of each cell type were determined after counting 300 cells (Locke et al. 2007).

#### Assessment of biochemical laboratory of the BALF supernatant

Matching the technique of (Smith et al. 1985), the total protein contents were measured using commercial kit (Biodiagnostic).

The enzymatic activity of lactate dehydrogenase (LDH) was assessed using commercially available colorimetric kit (abcam, ab102526).

Proinflammatory cytokine TNFα and IL6 contents were assessed in the BALF supernatant applying ELISA kits that was commercially available (R&D Systems, MN, USA).

Detection of Transforming Growth Factor β1(TGF-β1) levels in the lung tissues homogenates by ELISA kits purchased from Cusabio (Wuhan, P.R. China, catalog # CSB-E04727r).

#### Assessment of blood glucose levels

A blood sample obtained from rat tail on 8^th^ day was used for measuring blood glucose level by colorimetric method (Trinder 1969), using commercial kit (Biodiagnostic).

#### Assessment of Nrf2 and NFκB proteins levels in lung tissues homogenates

Protein levels of both nuclear factor (erythroid-derived 2)-like 2 (Nrf2) and nuclear factor kappa B (NF-κB) were measured in the homogenates of lung tissues by ELISA using commercially available kits from (MyBioSource.com; Nrf2: Cat # MBS012148 and NF-κB: Cat #MBS453975).

#### Assay of oxidative stress and antioxidant biomarkers in lung tissues homogenates

Lipid peroxidation marker malondialdehyde (MDA) level was evaluated using rat sandwich ELISA kit according to the manufacturers’ instructions (MyBioSource, CA, catalog # MBS727531).

Reduced glutathione (GSH) was assayed in lung tissue homogenate, according to (Ellman 1959).

Total antioxidant capacity (TAO) capacity was assessed using colorimetric method as described by manufacturer (Sigma Co., Cat. NO. MAK187).

#### Estimation of protein levels of phosphorylated phosphoinositide 3-kinase (PI3K), phosphorylated protein kinase B (Akt) and phosphorylated Forkhead box protein O1 (FoxO1)

Samples (≈20 μg) of identical protein concentrations were exposed to electrophoreses with 10% sodium dodecyl sulfate/polyacrylamide gel (SDS/PAGE) afterwards they were electro-transported to polyvinylidene difluoride membranes. Later, we blocked these membranes with (w/v) skimmed milk powder (5%) in PBS/Tween-20 at room temperature along two-h period. Afterwards, we incubate these membranes with polyclonal antibodies (1:1000): p-Akt (Thr450) was obtained from [Thermo Fisher Scientific, catalog number # PA5-37,469, RRID AB_2554078], p-PI3K (Tyr607) from [Thermo Fisher Scientific, catalog number # PA5-38,905, RRID AB_2555497] and p-FoxO1 (Ser249) attained by [Thermo Fisher Scientific, catalog number # PA5-64,676, RRID AB_2661988]. Then they were diluted in tris-buffered saline-tween comprising 1% bovine serum albumin and β-actin as internal control (Santa Cruz Biotechnology) diluted 1:1000 in blocking buffer. Afterwards, we incubate the membranes for one hour at room temperature with the corresponding secondary antibodies. Then they were washed and consequently developed. Lastly, photos of revealed protein bands were taken on the BioMax film (Kodak). The densitometrical quantification was performed using Image J software (Bio- Rad, California, USA). The bands densities were standardized against the corresponding density of the internal control (β-actin).

### Histological examination

#### Histological evaluation of the lung histoarchitecture changes by H&E stain

The neutral buffered formalin fixed lung tissues were first subjected to dehydration in ascending alcohol grades. Then xylene clearing and embedding in paraffin wax to form the paraffin blocks. We cut the tissue into slices with 5 μm thickness. These sections were mounted on slides to be stained with hematoxylin and eosin (H&E) for the routine histopathological inspection of general histoarchitecture of the lungs. 

#### Histological evaluation of lung fibrosis by mallory's trichrome stain

The paraffin blocks of formalin-fixed lung specimens were utilized in Mallory's trichrome staining of the collagen fibers.

#### Immunohistochemical analysis for assessment the alveolar macrophages (CD68) and inducible nitric oxide synthase (iNOS) proteins

The paraffin blocks of formalin-fixed lung specimens were utilized in immunohistochemistry. It was performed using the avidin–biotin–peroxidase method for discovery of alveolar macrophages (CD68) as well as expression of inducible nitric oxide synthase (iNOS). All samples were managed routinely. In Brief, 4-μm thickness paraffin sections were dewaxed in xylene. After that they were rehydrated in descending series of ethanol and subsequently immersed in 0.3% H2O2 for half an hour in order to block the endogenous peroxidase. Samples were microwaved of for fifteen min in citrate buffer (with pH 6) then the antigens were detected. To block non-specific binding, 10% goat serum was applied for half an hour. Later, tissue slices were washed mildly with PBS and the slices were incubated with iNOS antibody attained from (rat monoclonal antibody,1:500 dilution, Transduction Laboratories, San Diego, California, USA) and with CD68 antibody from (mouse monoclonal antibody, 1:200 dilution, code NCL-L-CD68; Leica Biosystems, Benton La, Newcastle Ltd, UK) overnight at 4 °C. Lastly, the slides were counterstained with Mayer’s hematoxylin then dehydrated and lastly mounted with DPX. Negative controls were attained by ignoring the incubation with the primary antibody. Meanwhile, positive immunoreactivity for both CD68 and iNOS staining was microscopically identified by visualization of brownish discoloration of the immunoreactive cells.

#### Ultrastructure examination of the lung

The right lobe that fixed in glutaraldehyde buffer and divided into small pieces (1 mm3 thickness), placed in phosphate buffer for 24 h, post-fixed in 1% osmium tetra-oxide, dehydrated and embedded in resin. The specimens were trimmed, followed by sectioning into semi-thin and ultrathin sections. Afterward, the semi-thin Sects. (1 μm) were stained with toluidine blue and examined with a light microscope.

The ultrathin sections were relocated to copper grids for staining with lead citrate and uranyl acetate. The sections were inspected by a transmission electron microscope at the electron microscope unit in Mansoura University using a Zeiss EM 100 S transmission electron microscope at 60 kV to determine the ultrastructure changes in the lung tissues.

#### Histomorphometrically assessment

We measured the subsequent quantitative morphometric parameters in the different experimental groups for the quantitative assay of pulmonary tissue: alveolar diameter (in toluidine blue stained sections), the mean area percentage of collagen fibers (in Mallory’s trichrome stained sections), the mean area percentage of the positive iNOS immunoreactivity and the positive CD68 immuno-expression (in immuno-stained slices). These parameters were measured in 5 non-overlapping and high-power perceptive fields from 6 rats of all studied groups by using the image analyzer computer system, (Leica Qwin 500, Microsystems Imaging Solutions Ltd, Cambridge, United Kingdom), in the image-analyzing unit presented in the Anatomy Department, Faculty of Medicine, Zagazig University, Egypt. Then we statistically analyzed all these morphometrically measured data. We measured the subsequent quantitative morphometric parameters in the different experimental groups for the quantitative assay of pulmonary tissue: alveolar diameter (in Toluidine blue stained sections), the mean area percentage of collagen fibres (in Mallory’s trichrome stained sections), the mean area percentage of the positive iNOS immunoreactivity and the positive CD68 immuno-expression (in immuno- stained slices). These parameters were measured in 5 non-overlapping and high-power perceptive fields from 6 rats of all studied groups. Briefly, using software image analyzer computer system, (Leica Qwin 500, Microsystems Imaging Solutions Ltd, Cambridge, United Kingdom), we applied the colour deconvolution to RGB colour images through H DAB matrices of the software. On DAB matrices, the images were transformed to 8-bit then the threshold was adjusted for DAB detection according to intensity. The threshold parameters were preserved constantly for all images. This process were performed in the image-analyzing unit presented in the Anatomy Department, Faculty of Medicine, Zagazig University, Egypt. Then we statistically analyzed all these morphometrically measured data.

### Statistical analysis

The data were represented as the means ± standard error of mean (SEM) and then compared using the one-way analysis of variance (ANOVA) in normally distributed variables, then followed by Tukey–Kramer multiple comparisons test. A level of probability (*P* value) ≤ 0.05 was considered significant. Our statistical analysis was performed using the statistical software program GraphPad Prism®, Version 5.00, for Windows (California, USA).

## Results

Following statistical comparisons among control and ALO groups, they showed non-significant variations; hence, all comparisons referred to the control group.

### ALO effect on blood glucose levels

**Fig. 2:**
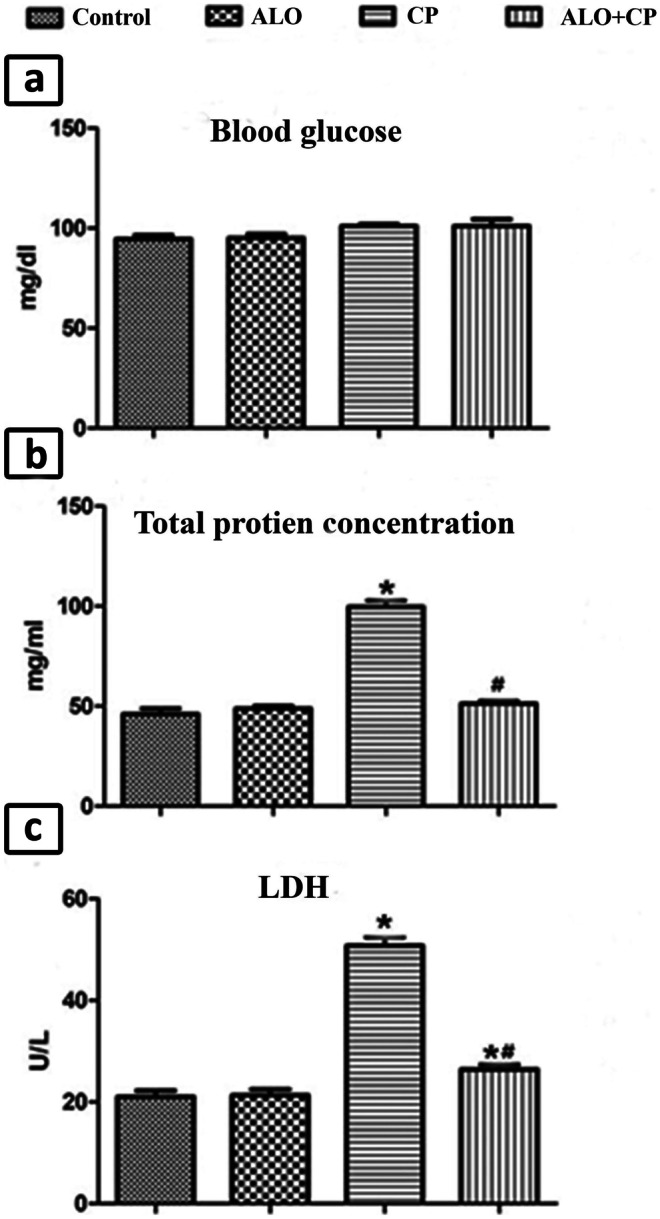
Representative image showing effect of alogliptin treatment on **a**. blood glucose level, **b**. total protein levels and **c**. LDH activitiy in BALF of CP-induced lung toxicity in rats. Data are represented as the means ± standard error of mean (SEM); analyzed by One-way ANOVA followed by Tukey’s multiple comparisons test. A level of probability (*P* value) ≤ 0.05 was considered significant. * Significant versus Control group; # significant versus CP-treated group. CP: cyclophosphamide; ALO: alogliptin; BALF: bronchoalveolar lavage fluid; LDH: lactic dehydrogenase

Comparison among all studied groups exhibited non-significant changes in blood glucose levels. Furthermore, all groups exhibited normal fasting blood glucose levels (less than 100 mg/dl) (Fig. 2a).

### The effect of ALO on the total and the differential leucocytic cells number

Single injection with CP in CP-treated group caused a significant upregulation in the total cells count and differential leucocytic cells number in BALF compared to untreated control group.

Meanwhile ALO pretreatment significantly decreased the count of total and differentiated leucocytic cells in ALO-treated rats compared to rats in CP-treated group. A significant decrease in the percentage of macrophages was observed in the ALO-treated group (Table 1).

### The effect of ALO on the total protein level and enzymatic activity of LDH in lung BALF

Single injection with CP produced significant rise in total protein content as well as enzymatic activity of LDH in rat lung BALF compared to control animals. Meanwhile, ALO pretreatment caused a significant decrease of total protein level and enzymatic activity of LDH compared to CP-treated group (Fig. 2b, c).

### Impact of ALO treatment on the pro‑inflammatory cytokines (TNF-α and IL-6) in rat lung BALF

**Fig. 3:**
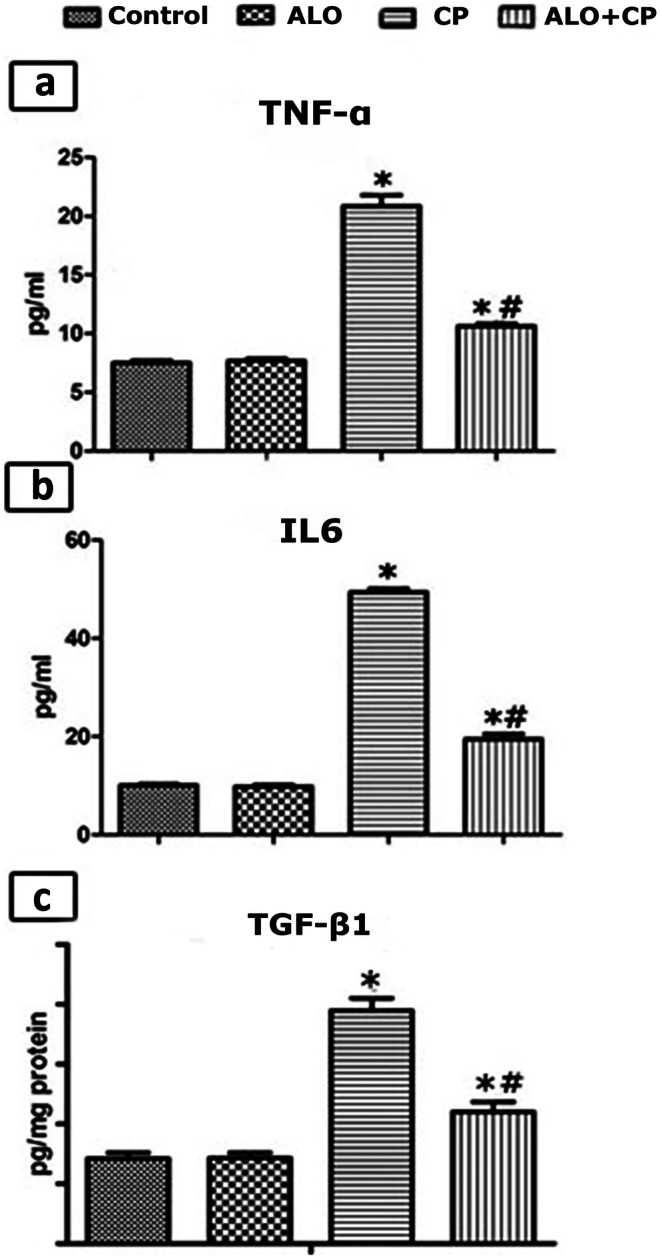
Representative image showing effect of alogliptin treatment on **a**.TNF-α and **b.** IL-6 in BALF and **c. **TGF-β1 levels in lung tissue homogenates of CP-induced lung toxicity in rats. Data are represented as the means ± standard error of mean (SEM); analyzed by One-way ANOVA followed by Tukey’s multiple comparisons test. A level of probability (*P* value) ≤ 0.05 was considered significant. * Significant versus Control group; # significant versus CP-treated group. CP: cyclophosphamide; ALO: alogliptin; BALF: bronchoalveolar lavage fluid; TNF-α: tumour necrosis factor alpha; IL-6: interleukin 6;TGF-β1: transforming growth factor

Concentrations of TNF-α as well as IL-6 were significantly raised in BALF of CP-treated rats compared to the untreated control rats. In ALO + CP-treated group ALO pretreatment caused a significant decline in the TNF-α, and IL-6 levels compared to CP-treated group (Fig. 3a, b).

### The influence of ALO on the TGF-β1 levels in the lung tissue homogenates

CP injection significantly increased the lung tissue level of TGF-β1as compared to control untreated group. Meanwhile, in ALO-treated group such effect was amended by ALO pretreatment which significantly declined the TGF-βl content compared to CP-treated group (Fig. 3c).

### The influence of ALO on the Nrf2 and NF-κB proteins contents in the pulmonary tissues’ homogenates

**Fig. 4:**
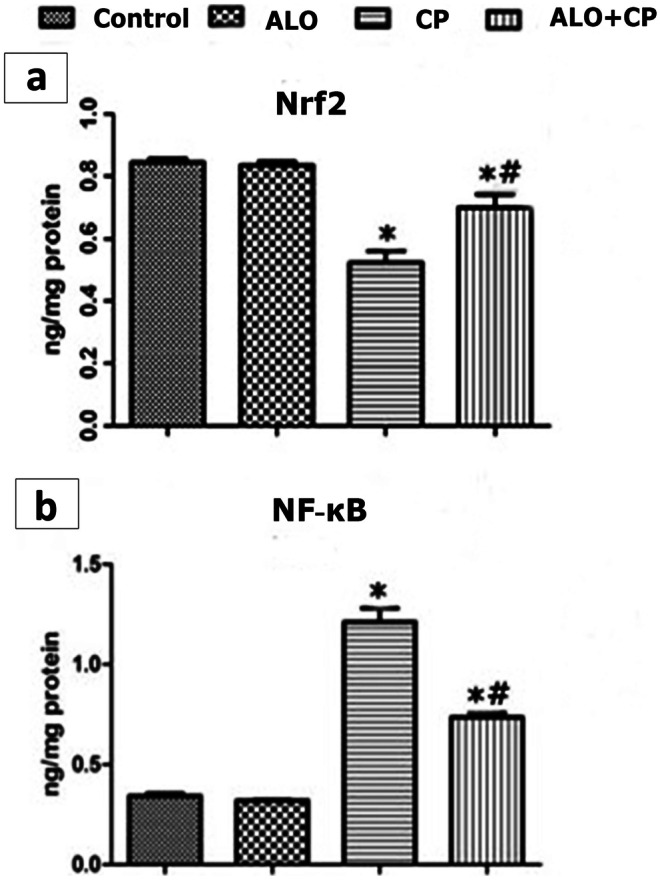
Representative image showing effect of alogliptin treatment on 1 **a.** **NF-kB** and **b.** Nrf2 levels in lung tissue homogenates of CP-induced lung toxicity in rats. Data are represented as the means ± standard error of mean (SEM); analyzed by One-way ANOVA followed by Tukey’s multiple comparisons test. A level of probability (*P* value) ≤ 0.05 was considered significant. * Significant versus Control group; # significant versus CP-treated group. CP: cyclophosphamide; ALO: alogliptin; NF-kB: Nuclear factor Kappa B; Nrf2: nuclear factor (erythroid-derived 2)-like 2

CP injection significantly decreased the Nrf2 level (Fig. 4a) compared to control normal rats. Inversely, NF-κB proteins levels (Fig. 4b) were significantly upregulated in CP-treated group compared to untreated Control rats. ALO pretreatment improved the aforementioned changes where Nrf2 level significantly increased and NF-κB proteins levels significantly decreased compared to CP-treated rats.

### Impact of ALO on oxidative stress markers in in the lung tissues homogenates

The lipid peroxidation marker, MDA, was significantly increased upon CP treatment compared to the untreated control group. Inversely, GSH as well as TAO were reduced significantly in CP-treated group compared to untreated control rats. AlO treatment improved these redox changes, where MDA was significantly decreased, while GSH and TAO were significantly increased as compared to the CP-treated rats (Table 2).

### AlO influence on protein content levels of lung p-PI3K, p-Akt and p-FoxO1

**Fig. 5:**
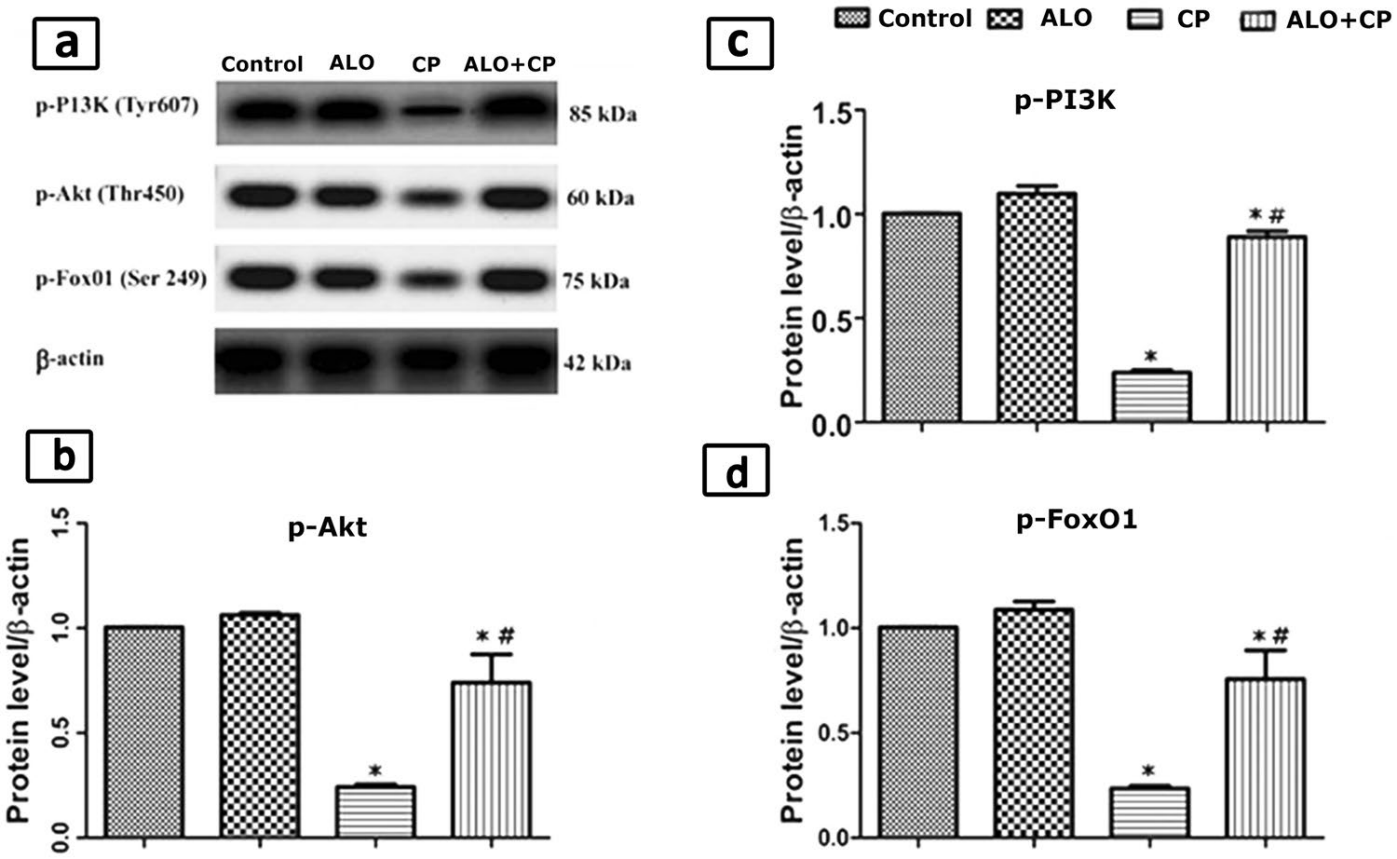
**a. **Western blot analysis of the lung tissue protein expression of phosphorylated phosphoinositide 3–kinase (PI3K), protein kinase B (Akt) and Fork head box protein O1 (FoxO1) in the whole cell lysates in CP-induced lung toxicity in rats. β-actin was used as an internal loading control Quantified expression of **b.** p-Akt; **c**. p-PI3K and **d**. p-FoxO. Data are represented as the means ± standard error of mean (SEM); analyzed by One-way ANOVA followed by Tukey’s multiple comparisons test. A level of probability (*P* value) ≤ 0.05 was considered significant. * Significant versus Control group; # significant versus CP-treated group. CP: cyclophosphamide; ALO: alogliptin

In CP-treated group the protein expression contents of p-PI3K, p-Akt and p-FoxO1 were significantly decreased as compared to the untreated Control rats. Preceding administration of AlO before CP significantly elevated protein concentrations of p-PI3K, p-Akt and p-FoxO1, as compared to the CP-treated rats (Fig. 5a, b, c, d).

### Histopathological findings of light microscopic inspection of the lung slices

#### Haematoxylin and eosin stain (H&E)

**Fig. 6:**
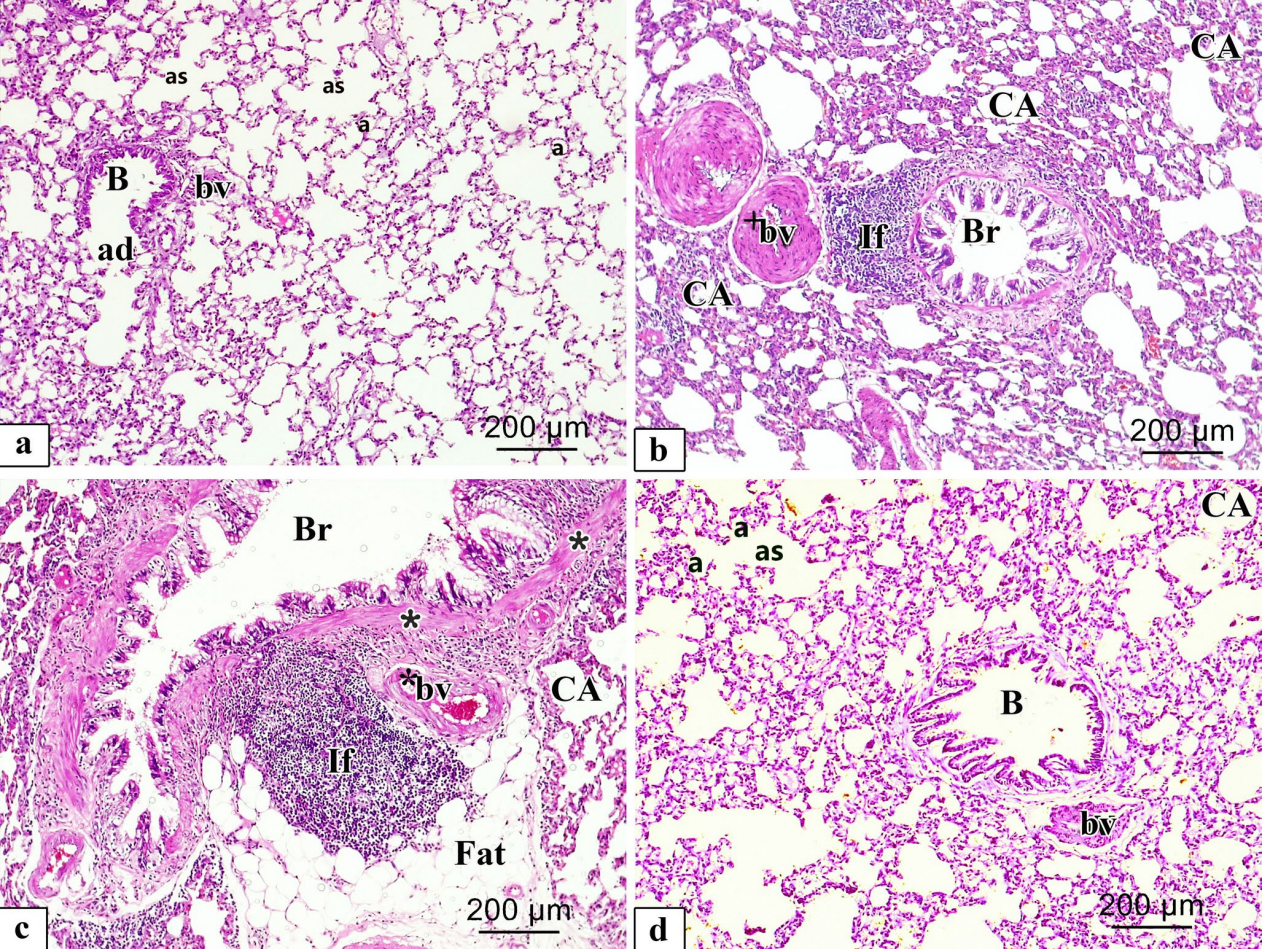
Representative microscopic images of H&E-stained sections of rat lung from different experimental groups. **a.** Control; **b**, **c.** CP and **d.** ALO + CP groups. large bronchiole (B), alveolar duct (ad), alveolar sac (as), alveoli (a), blood vessel (bv), collapsed alveoli (CA), Thick wall (*) bronchus (Br), extensive peri bronchial cellular infiltration (If), vascular congestion (*bv), thick wall blood vessel (+ bv), fatty cellular infiltrations (Fat)

Inspection of H & E-stained specimens of rat lung from the different experimental groups were shown in (Fig. 6a-d). The control group revealed lung tissue with normal spongy histological architecture and clear patent, different sized and polygonal alveoli divided by thin interalveolar septa, alveolar duct in addition to alveolar sacs, and a clear bronchiole mingled with the alveoli (Fig. 6a). However, lungs of CP group showed marked alveolar injury in the form of numerous collapsed alveoli, with severely thickened interalveolar septa and bronchi walls. Additionally, they showed heavy infiltration by inflammatory cells, fatty cellular infiltrations with thickened wall of the lung blood vessels (Fig. 6b, c). ALO recipient before the CP injection revealed a significant repair in histopathological lung architecture which seemed nearly the same as in the Control group, despite few thickenings of interalveolar septa in some regions with few collapsed alveoli with slightly thickened blood vessels could be observed (Fig. 6d).

#### Toluidine blue stain

**Fig. 7:**
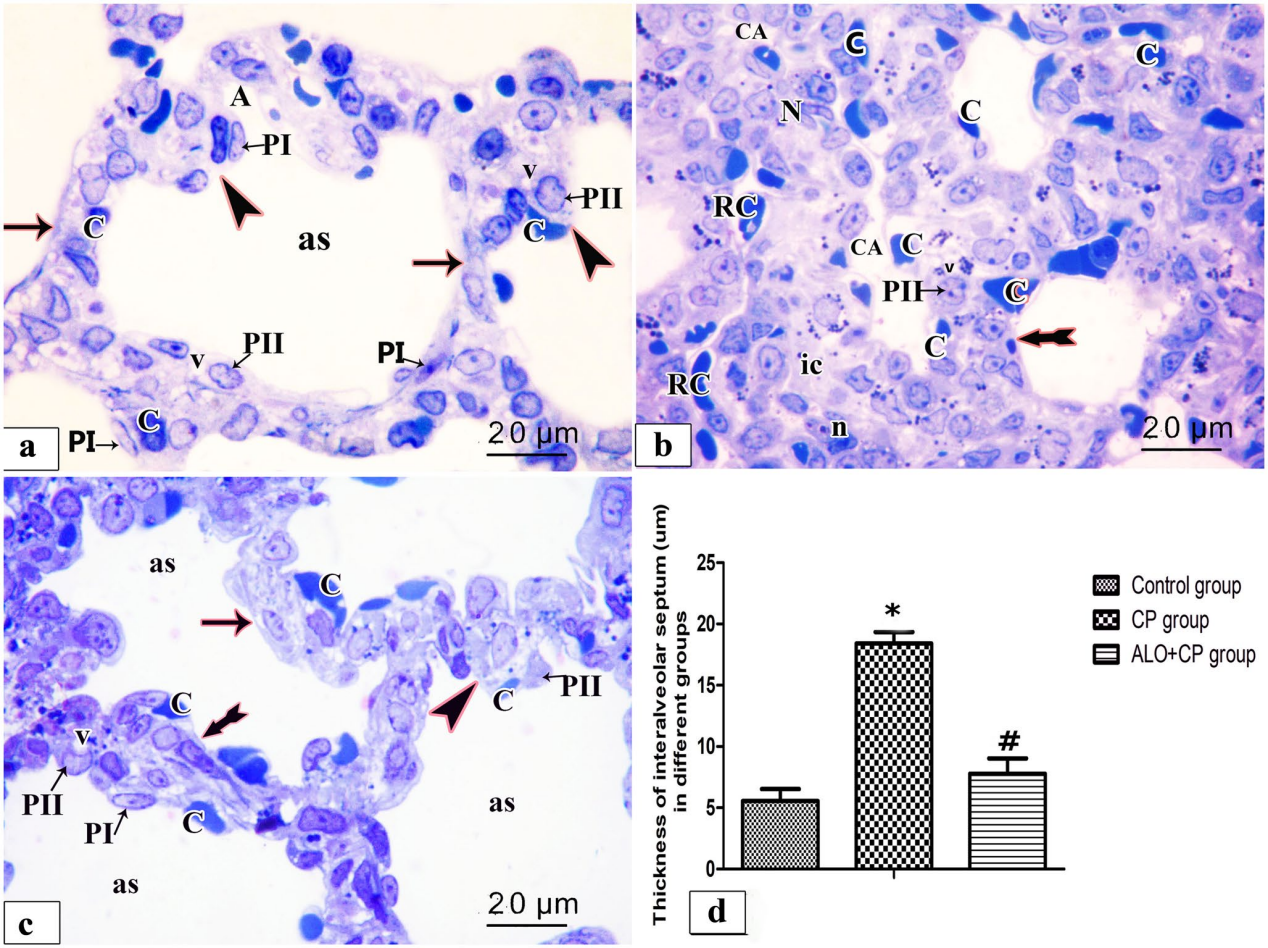
Representative microscopic images of semi sections of rat lung from the different experimental groups. **a.** control, **b.** CP, **c.** ALO + CP groups. Arrow, bifid arrow, and arrowhead illustrating primary interalveolar septa, thick primary interalveolar septa, secondary septa, respectively. alveoli sac (as), round nucleus of type II pneumocytes (PII), flat nucleus of type I pneumocyte (PI), vacuolated cytoplasm (v), single blood capillary (C), variable shapes interstitial cells (ic), irregular pale nuclei (N), dark oval nuclei (n). collapsed alveoli (CA), RBCs in the lumen (RC) **d.** Histogram shows the statistical analysis of the thickness of inter-alveolar septa from the different experimental groups. Data are represented as the means ± standard error of mean (SEM); analyzed by One-way ANOVA followed by Tukey’s multiple comparisons test. A level of probability (*P* value) ≤ 0.05 was considered significant. * Compared with the Control group, # compared with CP group

Semi sections examination of rat lung from the different experimental groups were shown in (Fig. 7a-d). Control rat lung revealed alveolar sac with primary interalveolar septa lined by type II pneumocytes with huge pale nuclei and vacuolated cytoplasm and type I pneumocyte with a well-defined flat shaped nuclei and single blood capillary facing the alveolar lumen. Secondary septa were formed of a group of cells; part of these cells at their tips have rounded nuclei with a vacuolated cytoplasm and single blood capillary facing the alveolar lumen whereas others have flat nuclei (Fig. 7a). Meanwhile, CP-treated rat lung, showed that the primary interalveolar septa are composed of variable shapes of interstitial cells, few of these cells have pale irregular nuclei with a vacuolated cytoplasm and others have dark oval nuclei. Various blood capillaries were obvious on sides of the alveolar wall and in the interalveolar septa. Note, secondary septa and pneumocytes type I are frequently observed (Fig. 7b). Lung of ALO recipient before CP treated showed most primary interalveolar septa lined by type II pneumocytes with large pale nuclei and vacuolated cytoplasm and type I pneumocyte with a flat shaped nuclei and single blood capillary facing the alveolar lumen. Secondary septa could be observed. Some primary interalveolar septa had interstitial cells of different shapes and double blood capillaries (Fig. 7c). Statistical results on the mean thickness of the primary interalveolar septa revealed significant rise in CP group (18.40 ± 0.947) compared to untreated Control rats (5.555 ± 0.986) and revealed a significant decline in ALO + CP group (7.787 ± 1.248) compared to CP-treated rats (*P* < 0.05) as presented in (Fig. 7d).

#### Mallory’s trichrome stain (MT)

**Fig. 8:**
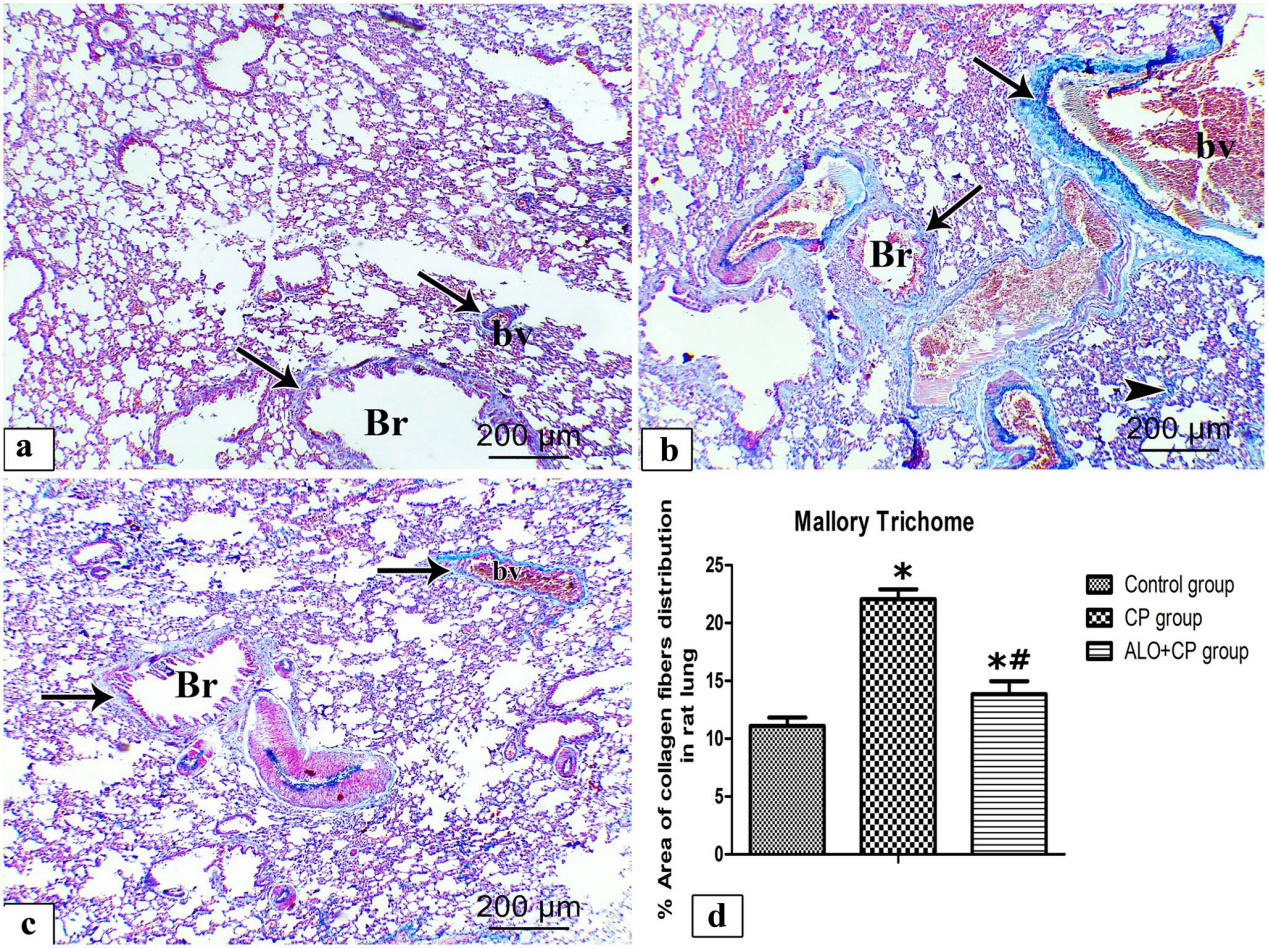
Representative microscopic images of Mallory’s trichrome stained rat lung sections from the different experimental groups. **a.** Control, **b.** CP and **c.** ALO + CP groups. Arrow refers to the blue staining of the collagen fibres around the bronchioles (Br), pulmonary blood vessels (bv). Arrowhead refers to the blue staining of collagen fibres within the lung interstitium. **d.** Histogram shows the statistical analysis of the changes in the % area of collagen fibres distribution from the different experimental groups. Data are represented as the means ± standard error of mean (SEM); analyzed by One-way ANOVA followed by Tukey’s multiple comparisons test. A level of probability (*P* value) ≤ 0.05 was considered significant. *Compared with the Control group, # compared with CP group

MT-stained rat lung sections from the different experimental groups were shown in (Fig. 8a-c). In lungs of Control rats, collagen normally distributed as fine fibers in the thin interalveolar septa, around pulmonary blood vessels as well as around bronchioles (Fig. 8a). CP-treated group revealed abundant collagen fibers distribution around the bronchioles, pulmonary blood vessels as well as within the lung interstitium (Fig. 8b). In contrast, ALO recipient before the CP injection decrease the condensation and distribution of the collagen fibers around bronchioles and pulmonary blood vessels as shown in (Fig. 8c). These results were confirmed `by morphometrically and statistically analysis of the % area of collagen fibers distribution that revealed a significant increment of the % area of collagen fibers of CP-treated rats (22.06 ± 0.852) compared to the untreated control rats (11.10 ± 0.718), in spite a significant decrement of % area of collagen fibers was observed in ALO + CP group (15.01 ± 0.776) compared to the CP-treated rats (*P* < 0.05) as presented in (Fig. 8d).

### Immunohistochemical results

#### Alveolar macrophage CD68 immuno-staining

**Fig. 9:**
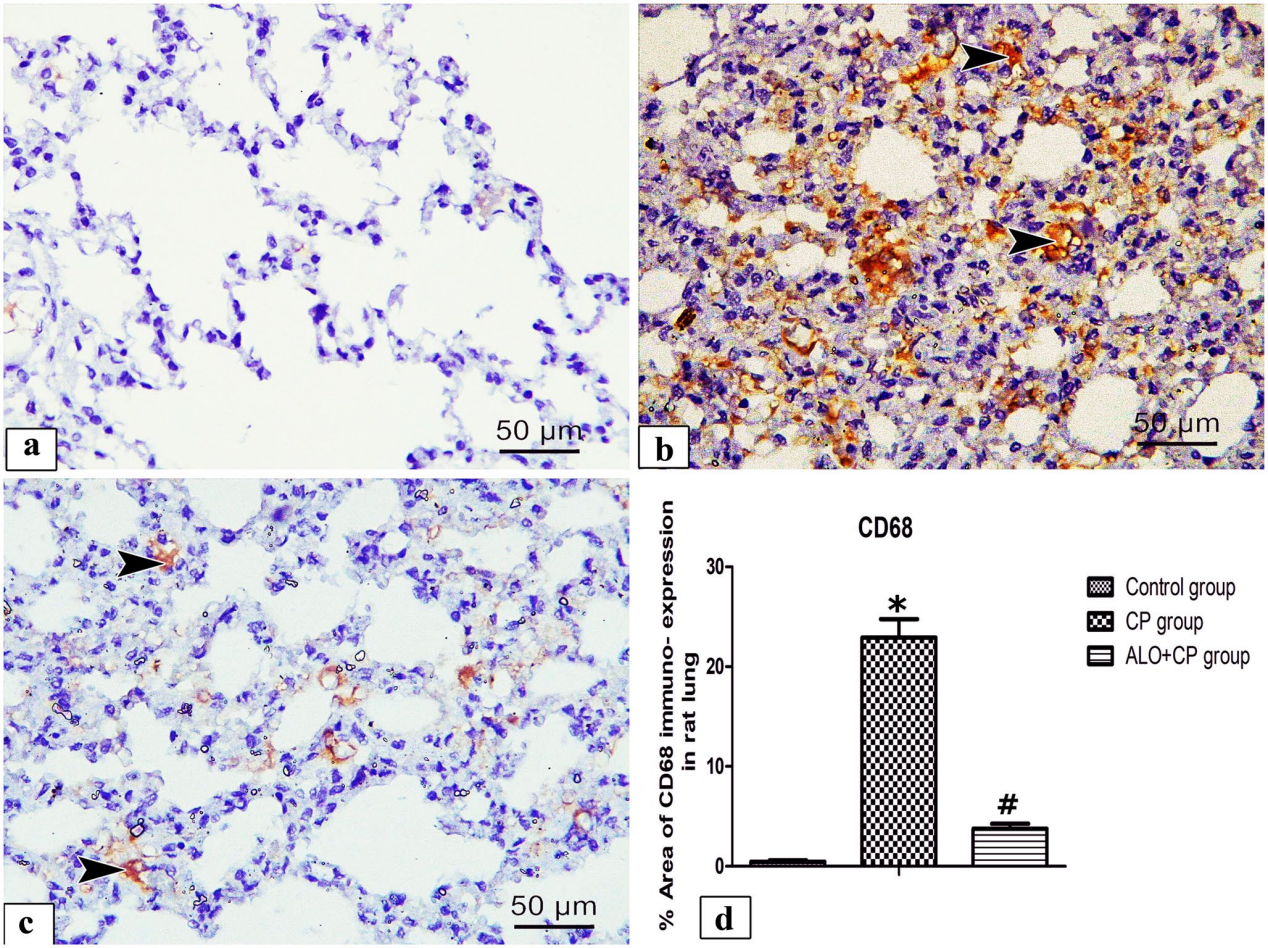
Representative microscopic images of immunohistochemically stained lung sections with anti- CD68 antibody of alveolar macrophage from the different experimental groups. **a.** Control, **b.** CP and **c.** ALO + CP groups. Arrowhead refers to positive brown cytoplasmic reacted cells. **d.** Histogram shows the statistical analysis of the of changes in the % area of CD68 immuno-expression from the different experimental groups. Data are represented as the means ± standard error of mean (SEM); analyzed by One-way ANOVA followed by Tukey’s multiple comparisons test. A level of probability (*P* value) ≤ 0.05 was considered significant. *Compared with the control group, # compared with CP group

Immunohistochemical staining of alveolar macrophage CD68 in lung tissues from different experimental groups were shown in (Fig. 9a-c). Lung of control group showed frequent positive brown cytoplasmic reacted cells (Fig. 9a). Although, Lung of CP group showed apparently positive cytoplasmic reaction in various cells (Fig. 9b). Inversely, lungs of ALO + CP group showed few brown positive cytoplasmic reacted cells (Fig. 9c). These results were confirmed morphometrically and statistically by analysis the % area of CD68 positive immuno-expression that revealed a significant higher expression of the CD68 in CP-treated group (22.50 ± 1.826) compared to control group (0.4715 ± 0.1369) (*P* < 0.05). These effects were significantly declined in rats supplemented with ALO before CP treatment (3.762 ± 0.355) (*P* < 0.05) compared to CP-treated group as presented in (Fig. 9d).

#### iNOS immuno-staining

**Fig. 10:**
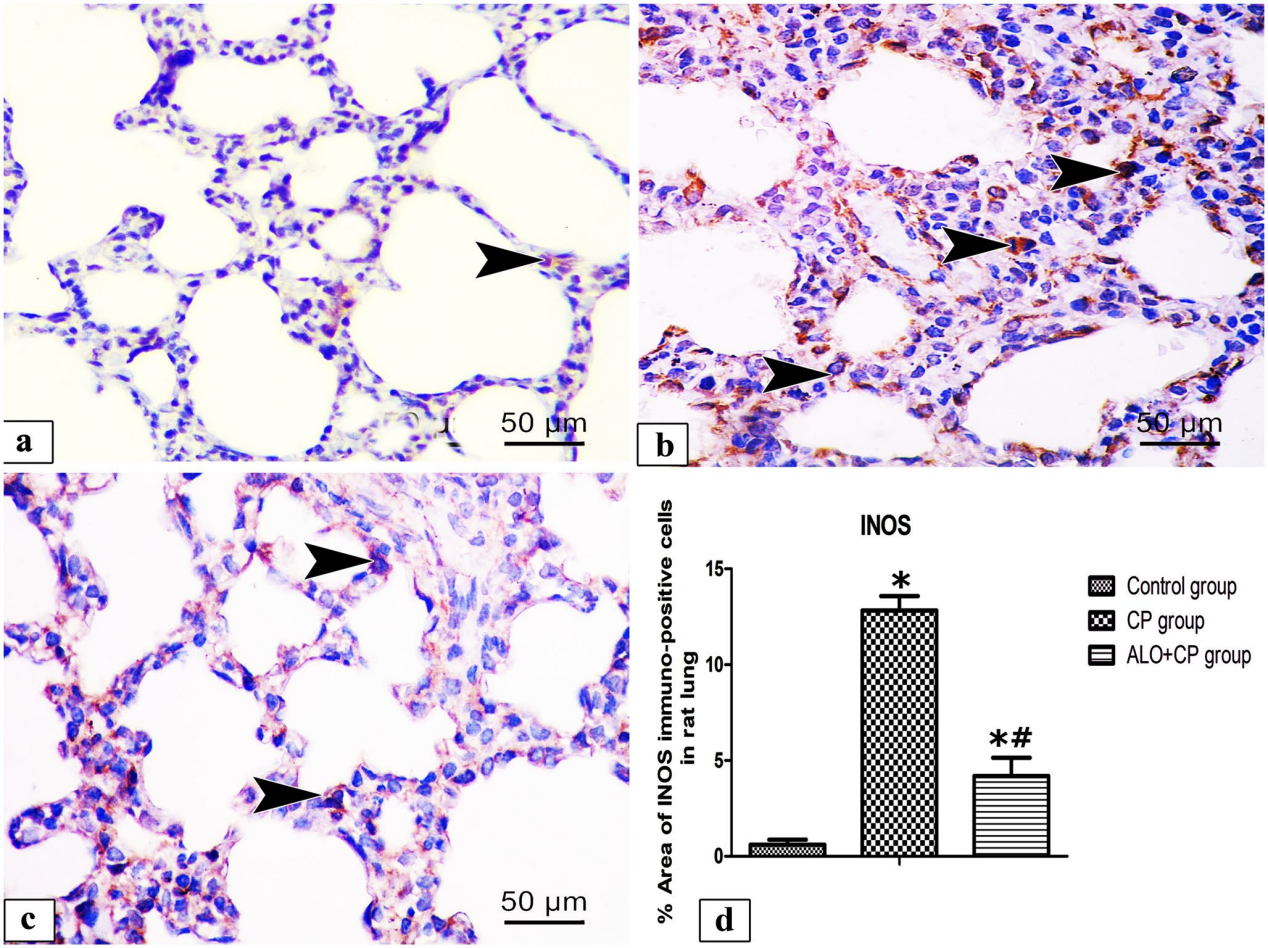
Representative microscopic images of immunohistochemically stained lung sections with anti- iNOS antibody from different experimental groups. **a.** Control, **b.** CP and **c.** ALO + CP groups. Arrowhead refers to the brown coloration of the immuno-positive cells. **d.** Histogram shows the quantitative and statistical analysis of the changes in the % area of immuno- positive cells from the different experimental groups. Data are represented as the means ± standard error of mean (SEM); analyzed by One-way ANOVA followed by Tukey’s multiple comparisons test. A level of probability (*P* value) ≤ 0.05 was considered significant. *P* < 0.05 * compared with the Control group, # compared with CP group

Immunohistochemical staining of iNOS in lung tissues from different experimental groups shown in (Fig. 10a-c). Lung of control group exhibited positive brown reaction in few alveolar cells (Fig. 10a). In contrary, lung of CP group showed apparently positive brown cytoplasmic reaction in numerous alveolar cells (Fig. 10b). On the other hand, lung of ALO recipient before CP group showed few positive brown cytoplasmic reacted alveolar cells (Fig. 10c). These results were confirmed `by morphometrical and statistical analysis of the % area of iNOS immuno-expression that revealed a significant higher expression of the iNOS in CP-treated group (12.83 ± 0.7576) compared with the untreated Control rats (0.6012 ± 0.2699). In ALO + CP group (4.195 ± 09,488), these effects were significantly declined compared to CP-treated rats (*P* < 0.05) as presented in (Fig. 10d).

#### Ultrastructural results of electron microscopic inspection of the lung specimens

**Fig. 11:**
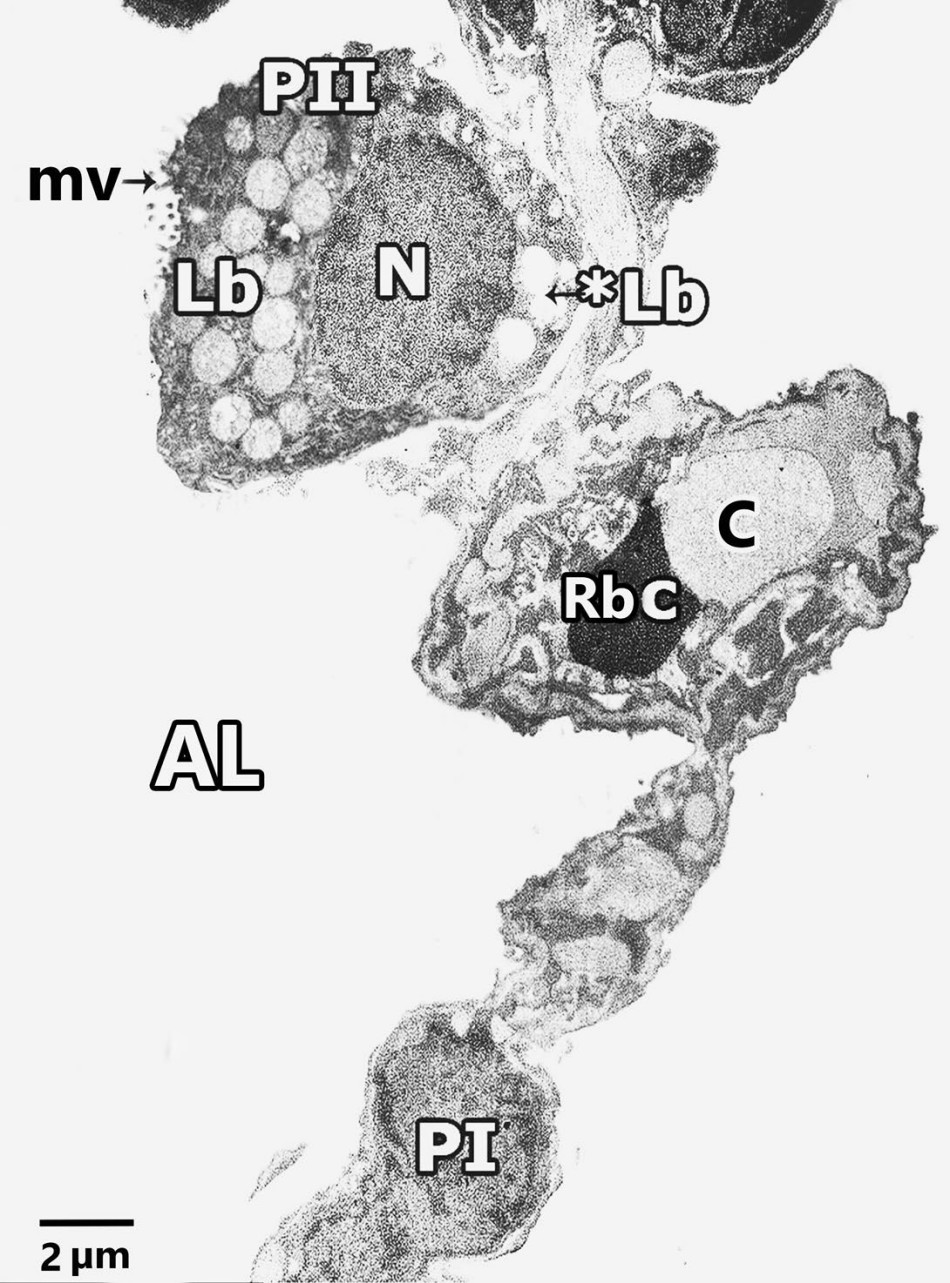
Representative transmission electron image of ultra-thin section of lung of control rat lung showing patent alveoli (AL) and thin interalveolar septa lined the alveoli with pneumocytes type I (P1), pneumocytes type II (PII) and single blood capillary (bc) contains Rbcs. Pneumocytes type II (PII) have large rounded euchromatic nucleus (N), short microvilli on its cell surface (mv) and full lamellar bodies (Lb) and empty lamellar body (*Lb) in its cytoplasm

**Fig. 12:**
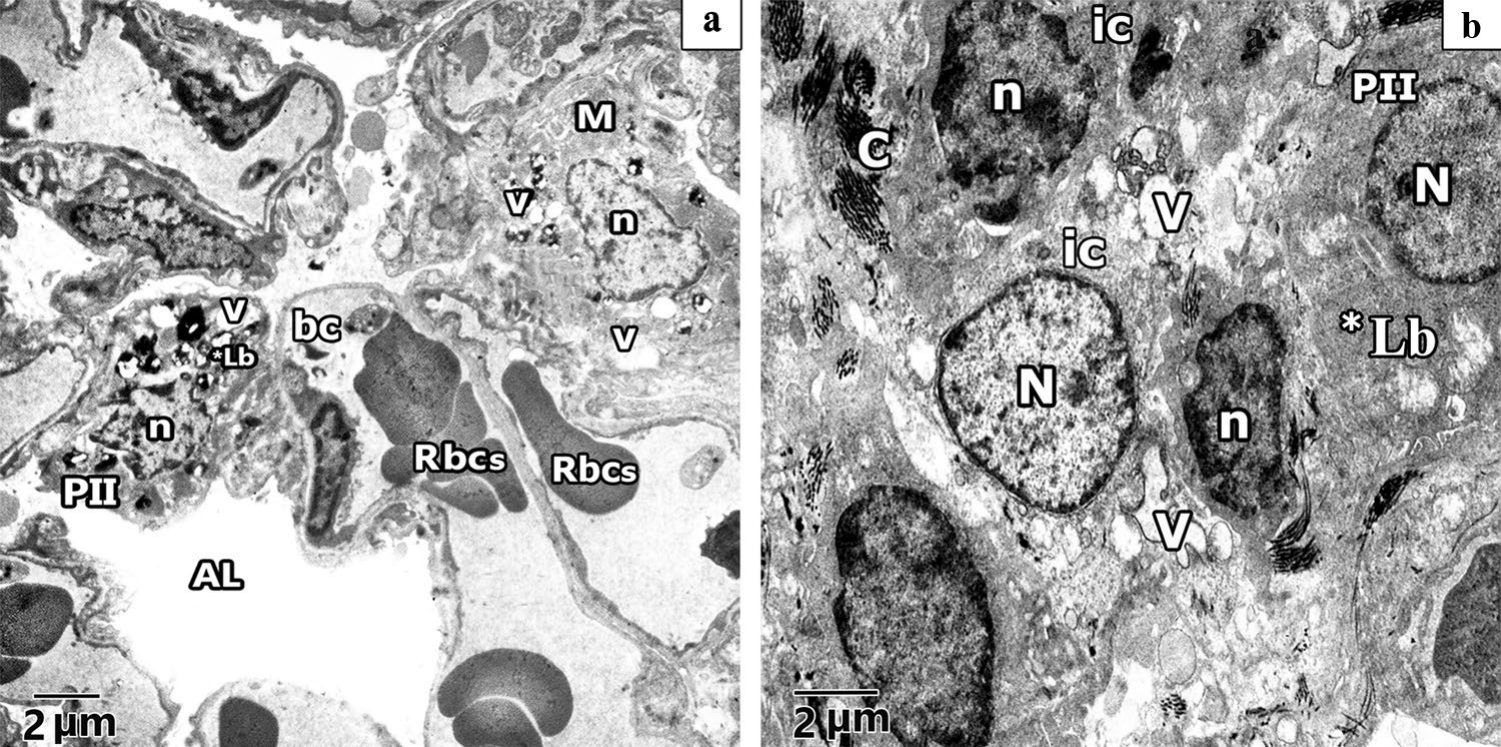
Representative transmission electron images of ultra- sections in CP rat lung, **a.** shows alveolar lumen (AL) lining with pneumocyte type II (PII) that have irregular heterochromatic nucleus (n) with degenerative changes of its lamellar bodies (*Lb) and atypical vacuolation (v). Macrophage (M) with irregular nucleus (n) and multiple vacuolation in its cytoplasm can be observed. Numerous congested blood capillaries (bc) containing red blood cells (RBCs) and RBCs inside the alveolar lumen (AL) can be noticed. **b.** shows thick interalveolar septa with multiple interstitial cells (ic), some has irregular elongated heterochromatic and dark stained nuclei (n) and other have round heterochromatic nuclei (N) with multiple vacuolation (V) and collagen fibres deposition. Pneumocyte type II (PII) with euchromatic nuclei (N) and degenerative changes of its lamellar bodies (*Lb) can be observed

**Fig. 13:**
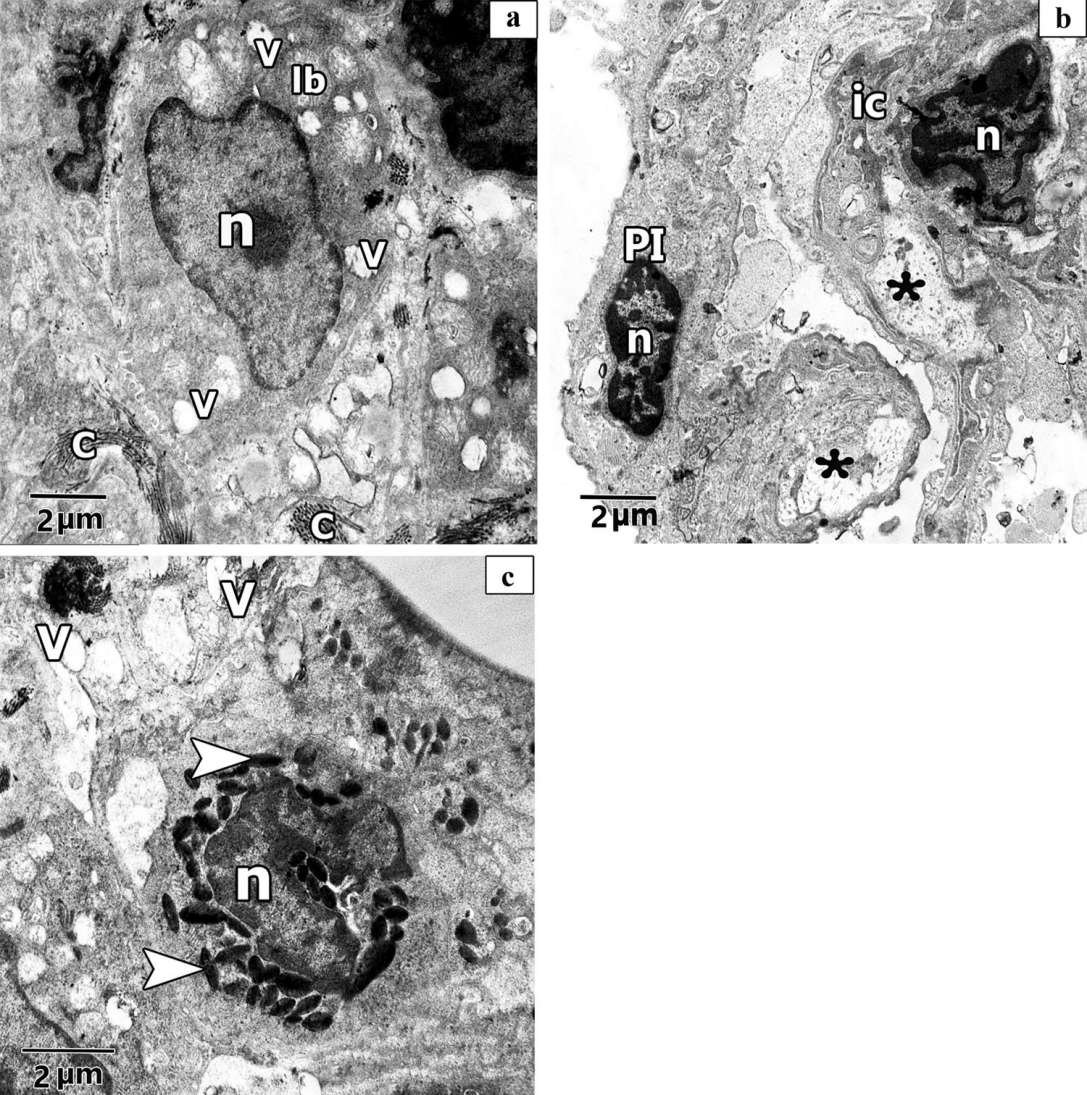
Representative transmission electron images of ultra- sections in CP rat lung shows **a.** other pneumocyte type II (PII) has irregular elongated heterochromatic nucleus (n) and atypical vacuolation (V) with degenerative changes of its lamellar bodies (Lb) leaving irregular empty vacuoles(V) in its cytoplasm associated with collagen fibres deposition (C). **b.** shows thick interalveolar septa with pneumocyte type I (PI) that has heterochromatic and dark stained nuclei (n). Interstitial cell (ic) with irregular elongated heterochromatic nucleus (n) can be observed. Lucent areas can be observed in the alveolar wall (*). **c.** shows eosinophilic cell with irregular heterochromatic nucleus (n) and electronic dense bodies as crystal like materials (arrowhead). Multiple vacuolations (V) can be observed

**Fig. 14:**
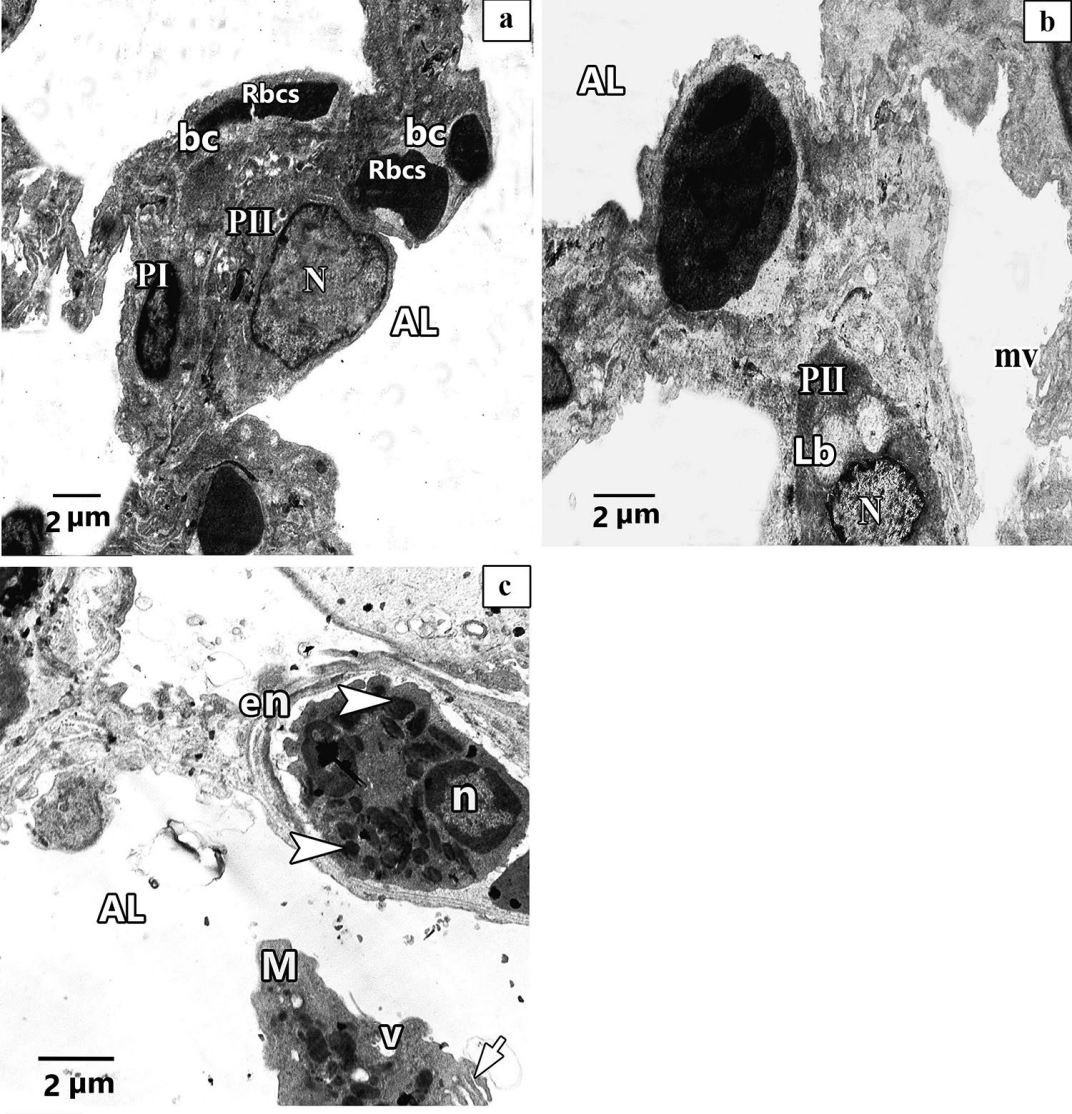
Representative transmission electron images of ultra-thin sections of ALO + CP rat lung. **a.** showing patent alveolar lumen (AL), apparent normal pneumocytes type II (PII) that have rounded euchromatic nucleus (N), pneumocytes type I (P1) have flat nucleus (N) and double blood capillaries (bc) contains red blood cells (Rbcs). **b.** Another magnification shows interalveolar septa with pneumocytes type II (PII) with rounded euchromatic nucleus (N) and full lamellar bodies (Lb) in its cytoplasm. Short microvilli on the cell surface (mv) can be observed. **c.** Another image shows interalveolar septa that have eosinophile (en) with shrunken nucleus (n) and electronic dense bodies (arrowhead). Exfoliated macrophage (M) with vacuoles (v) and pseudopodia (short arrow) can be seen in the alveolar lumen (AL)

Ultra-structurally examination of untreated Control group revealed patent alveoli lined by type I pneumocyte with flat nucleus and type II pneumocyte with a rounded euchromatic nucleus, various full and few empty lamellar bodies in its cytoplasm and short microvilli on its surface. Thin inter-alveolar septum containing single blood capillary. (Fig. 11a). Inversely, ultra-structural examination of alveolar architecture in CP-treated lungs revealed their damage. Pulmonary alveoli looked collapsed and predominantly lined by destructed pneumocytes type II. These cells showed atypical vacuolation with degenerative changes of their lamellar bodies causing irregular empty areas correlated with collagen fibres deposition. Thick interalveolar septa with multiple interstitial cells that had variable shapes nuclei can be noticed. Eosinophils, macrophages, and numerous congested blood capillaries can be observed in the inter alveolar septa (Figs. 12a, b, 13a, b, c). Administration of ALO with CP treatment ameliorated the destructive changes induced by CP (Fig. 14a, b, c).

**Fig. 15:**
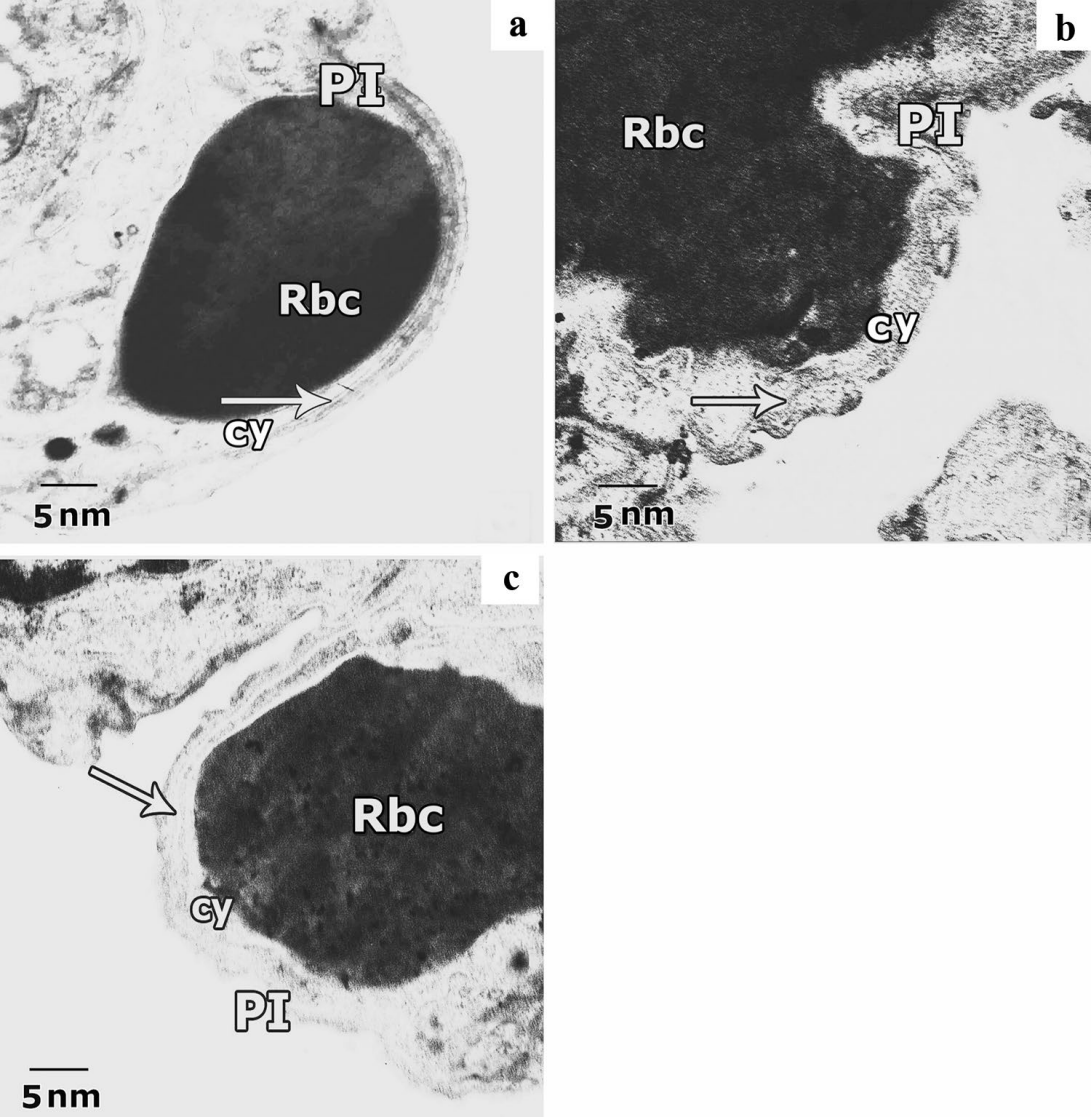
Representative transmission electron images of ultra-thin sections of rat lung from different experimental groups showing the air-blood barrier. **a.** Lung of the control group shows normal air-blood barrier, it is formed of the attenuated cytoplasm of pneumocyte type I (PI), fused basal lamina (arrow) and cytoplasm of capillary endothelial cells (cy) with red blood cell (Rbc). **b.** Lung of CP group exhibits deformed barrier, swollen cytoplasm of pneumocyte type I (PI), with irregularity in the fused basal lamina (arrow) and swollen cytoplasm of capillary endothelium (cy) with red blood cell (Rbc). **c.** Lung of ALO recipient group before CP reveals nearly normal barrier; capillary endothelium (cy) with red blood cell (Rbc), fuses basal lamina (arrow) and little swelling in cytoplasm of pneumocyte type I (PI) are observed

Ultrastructural examination of the air-blood barrier in the different studied groups, control group (Fig. 15a) revealed the identical layers that showed pneumocyte type I with attenuated cytoplasm beside the fused basal laminae of both endothelial cell and pneumocyte type I and capillary endothelial cells cytoplasm. CP-treated group exhibited deformed air-blood barrier with irregular and swollen cytoplasm of both pneumocyte type I and capillary endothelium (Fig. 15b). In the ALO + CP-treated group a nearly normal barrier but with little swelling in the cytoplasm of pneumocyte type I was noticed (Fig. 15c).

## Discussion

Cyclophosphamide (CP) is one of the most toxic cancer chemotherapeutics despite its high therapeutic effectiveness. Its limited use is due to its numerous organ damage and toxicity (Suddek et al. 2012; Ahlmann and Hempel 2016). Lung toxicity is a life-threatening complication of CP chemotherapy (Sun et al. 2014a, b). The toxicity of CP is produced mainly by its toxic metabolite acrolein that causes cellular apoptosis, encourage oxidative stress in addition to inflammation leading to lethal numerous organs injury (El-Kashef 2018). Hence, there is still a critical demand for highly protective adjunctive medications which can lessen the toxicity and enhance its therapeutic effectiveness.

Given the potent antioxidant, anti-inflammatory and antiapoptotic properties of alogliptin, as well as its proved efficacy against several animal models of CP-induced toxicity (Kabel 2018; Salama et al. 2020a, b, c). This study investigated its protective potential against CP pulmonary toxicity, emphasizing the potential contribution of PI3K/Akt/ FoxO1 and Nrf2 signaling pathway. In addition, to the top of our knowledge, this might be the first experimental study pointing the potential protective mechanism of ALO in pulmonary toxicity caused by CP and pointing the role of PI3K/Akt/FoxO1 axis.

Treatment with CP in this current study significantly demonstrated a marked increase in oxidative stress, inflammation, fibrosis, and apoptotic markers as well as deterioration in the histopathological pulmonary architecture. These hazardous effects were significantly ameliorated by ALO treatment.

Our results showed that CP-treated rats reported a marked enhancement in the oxidative burden status and worsening in the antioxidant defense mechanisms as depicted by significant rise in the lipid peroxidation marker MDA along with upregulation of iNOS protein that confirmed immunohistochemically by a significant increase in area percentage of iNOS immune-positive cells. This flaring of the oxidative stress status was reflected by a significant decrease in the antioxidants defense manifested by marked decrease in the Nrf2, glutathione (GSH) and TAO capacity.

The CP-generated oxidative stress caused by reactive oxygen species (ROS) production trigger the direct damage to pulmonary vascular endothelial cells (Chakraborty et al. 2009). This was in alignment with former clinical studies in which CP was able to induce chronic obstructive pulmonary dysfunction by inducing oxidative burden in addition to deteriorating the antioxidant protective pathways (Ismail et al. 2015; Sun et al. 2014a, b). In the same line El-Kashef., reported that CP administration upregulated the levels of MDA leading to disturbances in the physiological functions of other receptors and enzymes leading to decreased GSH contents (El-Kashef 2018).

In agreement with our results many studies reported that nitric oxide possesses a crucial role in CP-induced injury by upregulating iNOS protein (Al-Yahya et al. 2009; Mahmoud & Al Dera 2015). Nitric oxide by interacting with superoxide anions produce peroxynitrite (McKim et al. 2003) and activate NF-κB leading to increased pro-inflammatory cytokines production (Matata and Galiñanes 2002).

The Nrf2 is an intracellular antioxidant protective marker that is extensively distributed in numerous organs such as the lungs in which cascades of antioxidation and detoxification are habitually processed (ALHaithloul et al. 2019). Furthermore, the Nrf-2 contents in lung tissues demonstrated a close association with the susceptibility, seriousness, and healing of pulmonary diseases (Liu et al. 2019).

Our results reported a drop in Nrf2 signaling in CP-intoxicated animals and these in alignment with the previous studies (Aladaileh et al. 2019; ALHaithloul et al. 2019). Additionally, (Ahmed et al. 2017; Satta et al. 2017) reported that, The Keap1/Nrf2 signaling pathway has recently been nominated to be a redox-sensitive master regulator pathway through which Nrf2 dissociates from Keap1 under oxidative stress, then translocate into the nucleus where it binds the antioxidant response element (ARE), resulting in encouraging the expression of numerous cyto-defensive genes leading to antioxidant and anti-inflammatory reply.

Moreover, our results revealed that CP enhanced the concentrations of both LDH enzymatic activities and total proteins in lung BALF in comparison with the untreated control rats suggesting cell damage and loss of the alveolar wall integrity, airway cell influx in addition to microvascular leakage as stated by (Suddek et al. 2012). ROS and free radicals released due to CP exposure in rat lung caused their discharge from the cytoplasm into the blood vessels following breakup of the plasma membrane and cell damage (El-kashef 2018). Additionally, the increased MDA level leads to increased Ca^2+^ permeability across cell membrane, disrupts the alveolar capillary membranes, destructs the membrane proteins and enzymes, therefore permitting proteinaceous fluid leakage inside the pulmonary tissue producing cellular destruction and thus growing oxidative burden (Abdel-Latif et al. 2020; Nagaraj et al. 2012).

Fortunately, ALO treatment caused marked elevation of the antioxidants Nrf2, TAO and GSH levels along with significant reduction in the MDA level with down regulation of the iNOS protein confirmed by significant decline in the area percentage of immune-positive cells for iNOS. Additionally, ALO decreased the BALF total protein levels and LDH enzymatic activity. These findings implicate that ALO deteriorates the oxidative stress induced by CP toxicity. In alignment with our results Salama et al. stated that ALO hampered the oxidative stress induced by CP through reducing level of MDA lipid peroxidation marker and increasing the antioxidant enzyme GSH (Salama et al. 2020a, b, c). Additionally, Zhang et al. reported the same results in diabetic rats treated with ALO (Zhang et al. 2017). Kabel et al. reported that ALO increased the antioxidant glutathione peroxidase in a rat model of doxorubicin-induced testicular toxicity and restored Nrf2 content (Kabel 2018). Helmstädter et al. attributed this antioxidant activity to GLP1 receptor activation; as GLP1 receptor agonists enhance the activity of the antioxidant defenses and downregulates the oxidative stress parameters (Helmstädter et al. 2020). Hence, our results about the effect of alogliptin that restored the Nrf2 content accompanied by significant rise in the activity of the antioxidant enzymes assured the importance of Nrf2. As it may be a valuable therapeutic objective to prevent lung injury caused by CP by increased antioxidant defenses and decreased oxidative injury, inflammation, and apoptosis in CP-intoxicated rats.

Furthermore, the PI3K/Akt/FoxO1 is another signaling axis that has been noted to hinder cell survival progression upon its inhibition (Wang et al. 2019; Sciarretta et al. 2014). AKT phosphorylate Fork head box protein O1 (FOXO1) which modify several cellular processes as inflammation, apoptosis, and autophagy. Generally speaking, activation of these pathways encourages cellular survival, while hampering proapoptotic pathways (Oellerich and Potente 2012; Athauda and Foltynie 2016; Liu et al. 2016). The influence of oxidative stress produced by CP in our study was depicted in the reduced activity of PI3K and Akt signaling molecules along with reduced phosphorylated levels of FoxO1. In accordance with our results, the study of Albayrak et al. reported the repression of PI3K, Akt expression in the renal tissues of mice cured with CP (Albayrak et al. 2018). Moreover, earlier studies have clarified the repressive effect of the anticancer medications on the PI3K/Akt axis mediating oxidative stress occurring with these drugs (Zhu et al. 2009; Sun et al. 2014a, b).

Provided the responsibility of the PI3K/AKT/FoxO1 axis in maintaining lung function and structure and its repression by CP, it was fascinating to know whether the modulation of this pathway had a role in the pulmonary protective potentials of ALO. Our results covered the positive effect of ALO on the PI3K/Akt/ FoxO1signaling axis where it upregulated the lung tissue levels of PI3K, Akt and FoxO1 in normal and CP-ALO cured rats. The forementioned findings clearly support the notion that stimulation of the PI3K/Akt/ FoxO1 signaling paths have a crucial role in the protective effect of ALO against CP-induced lung toxicity. These results were in the same line of those of Salama et al. who proved that ALO caused upregulation in the PI3K/Akt/ FoxO1signaling pathway following its downregulation by CP in a rat model of hepatotoxicity (Salama et al. 2020a, b, c).

Athauda and Foltynie stated that PI3K/protein kinase B (AKT) pathways is an important downstream target of GLP-1 signaling (Athauda and Foltynie 2016). Additionally, Zeng et al. reported that the PI3K/Akt signaling pathway activation attenuates the oxidative stress by activating Nrf2 signaling, resulting in upregulation of antioxidants (Zeng et al. 2019).

The function of the inflammatory reactions in tissue harm caused by CP is properly recognized and the enhanced levels of the end markers of inflammation and fibrosis explain tissue damage induced by CP (Hammouda et al. 2020). Coinciding with our results many studies found that CP increased TNF-α, IL-6, NF-κB and TGF-β1 in the CP cured animals (Grynberg et al. 2017; Hammouda et al. 2020) which sequentially provokes serious inflammatory outcomes causing considerable cellular damage.

Furthermore, in the same line with our results, Freudlsperger et al. showed that CP toxicity initiate a significantly upregulated inflammatory processes due to a crosstalk between all TGF-β1, TNF-α and NF-κB signaling axis’s. The noteworthy increased TGF-β1 and TNF-α levels encourages NF-κB gene expression causing augmentation of the inflammatory process (Freudlsperger et al. 2012).

We observed remarkable inflammatory cellular infiltration which strongly supports the inflammatory paradigm of CP on the pulmonary tissues. ROS elicit an inflammatory surge by induction of numerous pro-inflammatory cytokines (NF-κB,TNF-α and IL-6) leading to neutrophil stacking to lung capillaries along with macrophages extravasation into the alveolar space and accompanied by leucocytes sensitization and infiltration (Giebelen et al. 2007; Abdelaziz et al. 2015).This was mirrored by the marked elevation in total and differential leucocytic number as well as LDH enzymatic activity in our results, and was approved with the marked upregulation in the number of CD68 positive macrophages in lung alveoli in CP-treated animals. The increased count of neutrophils, lymphocytes, macrophages, and eosinophils in lung BALF is linked to the inflammatory pathways caused by CP injection. Koyama et al. reported that neutrophils and macrophages have essential role in converting molecular oxygen to ROS (Koyama et al. 2001). Additionally, stimulated neutrophils released various inflammatory substance as TNF-α and ROS that additionally recall numerous inflammatory cells increasing the localized damage (Zhai et al. 2004). These results confirmed the highly significant rise in the thickness of inter-alveolar septum with CP-treated group compared with that in the untreated Control rats. This was in line with Al-Salih et al. who stated that CP-induced lung lesion characterized by thickening of alveolar wall with inflammatory cells infiltration (Al-Salih et al. 2020).

Additionally, many studies reveal the crosstalk among the Nrf2 and NF-κB paths (Ahmed et al. 2017). Nrf2 inhibit NF-κB activity in normal condition but in CP toxicity, decreased level of Nrf2 accompanied with increased NF-κB level as demonstrated in our results. This supports that the beneficial effect of Nrf-2 against CP toxicity is accomplished by two pathways; activation of antioxidant apparatus alongside the repressive action of NF-κB mediated proinflammatory pathways (Li et al. 2008).

The continuous surge of pro-inflammatory cytokines created a chronic condition of unlimited inflammation, proliferation, and fibrosis thus, impeding the process of lung tissue repair (Wang et al. 2014). TGF-β is a critical participant in the fibrosis pathogenic sequence by the encouragement of collagen in addition to increase in hydroxyproline content. Furthermore, the vicious cycle of fibrogenesis is triggered via improved fibroblast stimulation, fibronectin production and obstructing proteases accountable for lysis of the extracellular matrix (He et al. 2015; Wang et al. 2016; Xue and Li 2018). This was witnessed in the current study with the obvious increase in the percentage of collagen fibers area of lung tissues stained with Mallory trichome in CP group; the same as reported by Al-Salih et al. (Al-Salih et al. 2020).

The current study proved the anti-inflammatory efficacy of alogliptin against CP-induced toxicity where it resulted in considerable reduction in TGF-β1, TNF-α, IL-6 and NF-κB levels compared to rats treated with CP alone.

In the same line, Akita et al. reported that ALO through embarrassment of mRNA expression of NF-κB and TNF-α can decrease the expression of several inflammatory chemokines (Akita et al. 2015). Yet, it was shown in the study carried out by Uchida et al. that ALO reduced TGF-β1 gene expression decreasing a progressive renal fibrosis model (Uchida et al. 2017). Thus, the reduction in TGF-β1 levels in response to ALO treatment can further decline the expression of inflammatory and fibrotic molecules, and hence cellular apoptosis (Fabregat and Caballero-Díaz 2018).

In accordance with Al-Salih et al., these biochemical deteriorations were reflected on the integrity of pulmonary alveoli that showed vascular congestion, bleeding, alveolar collapse with very thick inter-alveolar septa in addition to inflammatory and fatty cellular infiltration of rats’ lungs (Al-Salih et al. 2020). Additionally, in a line with Şengül et al. we further confirmed these results by ultra- structural examination of lungs that revealed polymorphonuclear cell infiltration (neutrophils and macrophages), destructive type pneumocytes, distorted air-blood barrier with irregular and swollen cytoplasm of both pneumocyte I and capillary endothelium (Şengül et al. 2017). It was confirmed that CP-induced alterations in epithelial cells and alveolocapillary permeability caused congestion and edema (Shokrzadeh et al. 2014). Moreover, Ashry et al., revealed that histopathological examination of lung specimens isolated from CP-treated rats showed congestion, damage and/or edema of interalveolar septa, neutrophilic and macrophages infiltration. It was demonstrated that congestion and edema may be due to the changes produced by CP in epithelial cell structure as well as alveolocapillary permeability (Ashry et al. 2013).

Rats treated concurrently with alogliptin before recipient of CP showed a minimal degree of lung damage, no inflammatory infiltration in lung, less thickening of the alveolar septa in all examined field. These findings were in accordance with Vavrinec et al. and Shi et al. who reported that ALO protected against CP-induced sclerosis by inhibiting TGF-β1-induction, consequently, leading to reduction of inflammatory and fibrotic markers, and improvement in the histopathological features (Vavrinec et al. 2014; Shi et al. 2015).

Collectively, our histopathological findings verified the above stated biochemical findings and proved that ALO effectively alleviates the obvious CP-induced pulmonary toxicity. Decreased inflammatory cell infiltration, considerable decline in macrophage number and the percentage area of iNOS expression in addition to decreased collagen fiber deposition. These ameliorative alterations caused recovery in the structure and function of endothelium of the blood vessels which sequentially succeeded to diminish the CP-induced congestion and extravasation of RBCs.

## In conclusion

The present study provided persuasive evidence for the protective effect of ALO against CP-induced lung toxicity by mitigating the oxidative, inflammatory, fibrotic, and apoptotic impact of CP. The pulmonary protective efficacy of ALO was linked to their capability to stimulate Nrf2 and PI3K/Akt/FoxO1 signaling pathways, resulting in reduction of oxidative stress, apoptosis, and tissue damage. Therefore, ALO might represent hopeful pulmonary protective drug in patients on chemotherapy, awaiting further investigations and clinical studies to discover the exact mechanism(s) underlying its favorable effects.

**Table 1:** Effect of alogliptin treatment on the total and differential leucocytic counts in the BALF pellets of all experimental groups

**Groups**	**Total cells count × 10**^**5**^	**Neutrophils %**	**Eosinophils %**	**Lymphocytes %**	**Macrophages %**
**Control**	4.54 ± 0.11	2.83 ± 0.088	0.52 ± 0.01	13.23 ± 0.17	81 ± 1.6
**ALO**	4.6 ± 0.14	2.86 ± 0.09	0.51 ± 0.01	13.1 ± 0.25	80.5 ± 1.39
**CP**	12.8 ± 0.3^***^	47.55 ± 1.1^***^	5.2 ± 0.05^***^	35.33 ± 0.17^***^	12.11 ± 0.77^***^
**ALO + CP**	4.6 ± 0.12^###^	9.8 ± 0.3^***###^	1.13 ± 0.03^***###^	9.6 ± 0.2^***###^	71.62 ± 2.06^**###^

Data are represented as the means ± standard error of mean (SEM); analyzed by One-way ANOVA followed by Tukey’s multiple comparisons test. A level of probability (*P* value) ≤ 0.05 was considered significant. * Significant versus control group; # significant versus CP-treated group. *CP* cyclophosphamide, *ALO* alogliptin, *BALF* bronchoalveolar lavage fluid

**Table 2:** Effect of alogliptin treatment on MDA, reduced GSH and TAO levels in lung tissue homogenates

**Groups**	**MDA levels** (nmol/mg protein)	**GSH** (nmol/mg protein)	**TAO** (mg/gm protein)
**Control**	7.7 ± 0.34	41.83 ± 1.87	26.5 ± 2.23
**ALO**	7.5 ± 0.34	40 ± 0.77	27.5 ± 1.9
**CP**	47 ± 1.67 *	17 ± 0.43 *	13.37 ± 0.42 *
**ALO + CP**	21.02 ± 2.16*#	31.3 ± 1.4*#	22 ± 0.7 #

Data are represented as the means ± standard error of mean (SEM); analyzed by One-way ANOVA followed by Tukey’s multiple comparisons test. A level of probability (*P* value) ≤ 0.05 was considered significant. * Significant versus control group; # significant versus CP-treated group. *CP* cyclophosphamide, *ALO* alogliptin, *TAO* total antioxidants, *MDA* malondialdehyde, *GSH* reduced glutathione

## Abbreviations

LDH: Lactate dehydrogenase
TNF-α: Tumor necrosis factor α
IL-6: Interleukin 6
MDA: Malondialdehyde
Nrf2: Nuclear factor (erythroid-derived 2)-like 2
TAC: Total antioxidant capacity
GSH: Reduced glutathione
NFκB: Nuclear factor kappa B
TGF-β1: Transforming growth factor β1
PI3K: Phosphoinositide 3–kinase
Akt: Protein kinase B
FoxO1: Fork head box protein O1
iNOS: Inducible nitric oxide synthetase
i.p.: Intraperitoneal
